# Digital instrument simulator platform to support the development of noninvasive optical NIR device for placenta monitoring

**DOI:** 10.1117/1.JBO.31.2.027003

**Published:** 2026-02-20

**Authors:** Charly Caredda, Frédéric Lange, Niccole Ranaei-Zamani, Uzair Hakim, Olayinka Kowobari, Dimitrios Siassakos, Sara Hillman, Anna L. David, Subhabrata Mitra, Ilias Tachtsidis

**Affiliations:** a INSA-Lyon, Université Claude Bernard Lyon 1, UJM-Saint Etienne, CNRS, Inserm, CREATIS UMR 5220, U1294, Lyon, France; b University College London, Department of Medical Physics and Biomedical Engineering, London, United Kingdom; c University College London, EGA Institute for Women’s Health, London, United Kingdom

**Keywords:** placental monitoring, near infrared spectroscopy, placental sensitivity, light propagation modelling

## Abstract

**Significance:**

Abnormal placental development is a major cause of adverse pregnancy outcomes, but current methods for placenta monitoring are not suitable for bedside use. Continuous-wave near-infrared spectroscopy (CW-NIRS) is an optical technique that takes advantage of the near-infrared light to provide functional measurements such as tissue oxygenation at the bedside. However, the placenta is an organ located beneath several layers of tissue, making robust measurement of placental oxygenation with a CW-NIRS device a complex task.

**Aim:**

We propose a framework based on light propagation simulations to evaluate the sensitivity of CW-NIRS devices for placenta detection, along with tools to support NIRS instrument development for engineers.

**Approach:**

The maternal abdomen was modeled as a four-layer structure (i.e., skin, adipose tissue, muscle, and placenta). We used a numerical solution of the diffusion equation using a finite-element method to assess the sensitivity to measure placental function under various conditions (tissue layer thickness, skin tone, tissue oxygen saturation). We used a calibration procedure to evaluate the probability of acquiring a sufficient irradiation with a CW-NIRS device. We collected ultrasound abdomen images from 142 healthy pregnant participants that we segmented and digitized to demonstrate our approach.

**Results:**

With a Mini-CYRIL CW-NIRS device, we showed that placenta monitoring is not possible when using short integration time with a subject having a deep placenta (≥20  mm) and dark skin tones. With an integration time of 10 s and a temporal binning of 10 points, simulations indicated that subjects with very fair skin tone have a placenta-scanning probability of 12% at a placenta depth of 20 mm and 39% at a depth of 10 mm, using a 50 mm source–detector separation. Thick skin and dark skin tones act as a filter on the NIRS signal, blocking backscattered light and leading to greater absorption in deeper tissues. The spatially resolved spectroscopy method can be used to monitor placental oxygenation with a placenta close to the surface and an oxygen saturation in the muscle layer lower than that of the placenta. The simulation of a realistic cohort of 142 maternal abdomens aimed to identify the optimal acquisition conditions for CW-NIRS devices to be used in placental monitoring.

**Conclusions:**

We proposed a framework to evaluate and optimize CW-NIRS sensitivity for placenta detection. Further work is needed to improve the reliability of placental tissue oxygenation.

## 1 Introduction

Abnormal placental development is a major cause of adverse pregnancy outcomes, such as hypertensive disorders, fetal growth restriction, and stillbirth.^1^^,^^2^ These adverse outcomes are associated with poor placental oxygen perfusion,^3^ which is challenging to assess during clinical practice. Ultrasound imaging with Doppler assessment of the umbilical artery and middle cerebral artery remains the primary clinical modality for monitoring fetal growth and well-being. These Doppler measurements provide fetal and placental blood flow data rather than direct oxygenation.^4^^,^^5^ Magnetic resonance imaging techniques can assess placental oxygenation,^6^^,^^7^ but these approaches are not suitable for bedside monitoring and are resource dependent.^8^ Photoacoustic imaging uses pulsed light to generate ultrasound waves in tissue, providing high-contrast, high-resolution images of tissue morphology and function. This technique has been extensively developed for preclinical imaging applications^9^ and can be used to study preclinical placental anatomy and function.^10^ However, clinical translation of photoacoustic devices has not yet been achieved due to limitations such as a lack of standardization and safety constraints.^11^

Near-infrared spectroscopy (NIRS) is an optical technique widely used to monitor deep tissue oxygenation and, in particular, brain oxygenation. These devices take advantage of near-infrared (NIR) light to monitor deep tissues such as the brain,^12^^,^^13^ muscles,^14^^,^^15^ and placenta.^16^^,^^17^ NIRS can measure oxy-hemoglobin (${\mathrm{HbO}}_{2}$) and deoxy-hemoglobin (Hb) concentrations and has been extensively employed to monitor tissue hemodynamics.^18^^–^^20^ NIRS techniques, including continuous-wave (CW), time-domain (TD), and frequency-domain (FD), offer diverse approaches for measuring tissue oxygenation and hemodynamics. Continuous-wave near-infrared spectroscopy (CW-NIRS) measures relative changes in light intensity, offering simplicity of operation. These instruments have several limitations, mainly the large variability in the absolute quantification of oxygenation.^20^ Time-domain NIRS provides precise depth discrimination by analyzing photon arrival times, and frequency-domain NIRS assesses both amplitude and phase shifts of modulated light, enabling quantification of absolute optical properties.^21^

Wang et al.^16^ developed a frequency-domain NIRS device to monitor placental oxygen saturation during pregnancy. Authors showed that NIRS measurements of placental oxygenation were correlated with adverse pregnancy outcomes and histopathological findings such as maternal vascular malperfusion.^16^ The NIRS device was integrated with ultrasound imaging to model light propagation in a multilayered medium. A multilayer analytical light-transport model was then fitted to measurements to quantify hemodynamics in the placenta layer.

Commercial brain oximeter CW-NIRS systems have also been used to monitor placental oxygenation. Nguyen et al.^17^ measured placental oxygen saturation using a CW-NIRS device (NIRO-300, Hamamatsu Photonics, Hamamatsu City, Japan). In their study, the authors demonstrated that higher tissue oxygenation indexes in pregnant women suggest a decline in placental function. The calculation of the oxygenation index is based on the spatially resolved spectroscopy (SRS) technique,^22^ which requires assumptions about tissue homogeneity. As the maternal abdomen comprises layered structures, the SRS algorithm does not allow accurate quantification of absolute ${\mathrm{HbO}}_{2}$ and Hb concentrations.^20^

Despite this limitation, CW-NIRS devices are adapted for placenta monitoring at the bedside because they are portable, low-cost, and easy to operate.^17^^,^^23^^–^^25^ They use continuous light sources and simple detectors, allowing real-time, noninvasive tissue monitoring. Unlike time or frequency-domain systems, CW-NIRS is compact and practical for clinical environments. Recently, miniaturized broadband CW-NIRS devices^26^ have emerged, allowing the quantification of hemoglobin oxygenation and oxidation of cytochrome-c-oxidase, a marker of mitochondrial function involved in more than 95% of cellular adenosine triphosphate production.^27^

Robust bedside monitoring of placental hemoglobin oxygenation and mitochondrial function with CW-NIRS is important as it could enable early detection of pregnancy complications.^16^^,^^28^ The placenta is an organ located beneath several layers of tissue, making robust measurement of placental oxygenation with a CW-NIRS device a complex task. The light collected by CW devices includes contributions from different layers up to ∼4.2  cm in depth^16^ (skin, adipose tissue, muscle, and placenta), but the proportions of these contributions cannot be determined from the measurements. Moreover, CW-NIRS quantification models rely on the assumption of a homogeneous tissue, which induces quantification errors.^17^^,^^23^^,^^24^

The use of CW-NIRS for bedside placenta monitoring raises two main questions: (1) What is the sensitivity for measuring placental function? (2) Can the collected signal be unmixed to accurately monitor placental function? In this work, we address the first question by presenting a dedicated framework. The second question will be addressed in future work.

In developing NIRS technologies for placental monitoring, tools and methodologies are required to investigate photon sensitivity and measurement accuracy within the multilayered abdomen. To better understand the possibilities and current limitations of CW-NIRS estimation of placental oxygenation, we developed a framework based on a numerical solution of the diffusion equation to evaluate the sensitivity of CW-NIRS devices for monitoring placental function. Using this framework, we proposed an approach to assess sensitivity for scanning tissue with an adjustable distance CW-NIRS device, employing a calibration procedure with a solid phantom. We evaluated the placental sensitivity of an in-house developed adjustable distance broadband CW-NIRS device (Mini CYRIL^26^) based on ultrasound measurements of skin, adipose tissue, and muscle layer thicknesses, as well as skin tones of 142 healthy pregnant participants. Mini CYRIL has not been used for clinical research studies to monitor the placenta, but we aim to use this framework in our future work. This framework can support the optimization of NIRS acquisition parameters (e.g., integration time, source–detector separation) during instrumentation development and before clinical application and identify study cases (e.g., tissue thickness, skin tone) suitable for monitoring placental function. This framework can also be used to study the effect of skin tones on CW NIRS measurements. Indeed, darker skin tones have a major impact on the detection of a sufficient amount of signal and on the sensitivity of the placenta. This work can also support the evaluation and improvement of algorithms for the quantification of absolute ${\mathrm{HbO}}_{2}$ and Hb concentrations.

## 2 Material and Methods

**Fig. 1:**
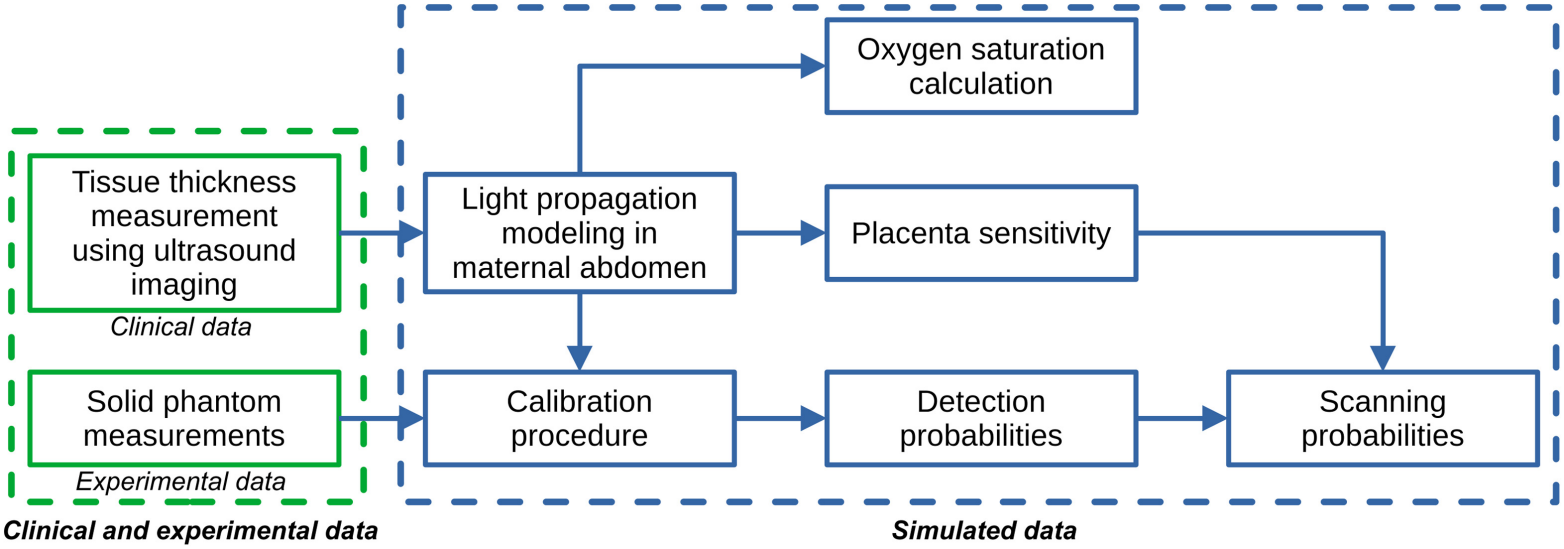
Flowchart of digital instrument simulator for evaluating the sensitivity of CW-NIRS devices in monitoring placental function.

In Fig. 1, we represented the flowchart of the digital instrument simulator for evaluating the sensitivity of CW-NIRS devices in monitoring placental function. The digital instrument simulator included clinical, experimental, and simulated data. Clinical ultrasound images were acquired on 142 healthy participants during pregnancy at the Institute for Women’s Health at University College London Hospital (UK). Ultrasound images were segmented and used for measuring tissue thickness and placenta depth (distance from skin to placenta). The diffusion equation was numerically solved using the finite element method to model the light propagation in the maternal abdomen and the acquisition of the detected light with an in-house developed adjustable distance broadband CW-NIRS device. With these simulations, we calculated the placenta sensitivity, which represents the probability of a CW-NIRS device scanning the placenta layer. With the simulated data, we calculated the probability of a detector of the CW-NIRS device to acquire a signal that is significantly above the noise level (detection probability). Simulated data were calibrated with experimental data measured on a solid phantom with a CW-NIRS device. Using simulated data, we evaluated the accuracy of adjustable distance CW-NIRS devices for measuring placenta oxygen saturation with the SRS algorithm.^22^

### 2.1 Light Propagation Modelling in the Maternal Abdomen

We used the Redbird software^29^^,^^30^ to simulate the propagation of light emitted in the maternal abdomen and its detection with the adjustable distance NIRS device Mini-CYRIL.^26^ Details about the Mini-CYRIL device can be found in Sec. 2.5. Redbird is an open-source MATLAB/GNU Octave toolbox designed for solving the forward and inverse problems for diffuse optics applications. The toolbox is based on a numerical solution of the diffusion equation using a finite-element method. It runs ∼100 times faster than GPU-based Monte Carlo MMC/MCX software^31^^,^^32^ and produces simulated radiative quantities free of stochastic noise. Redbird computes fluence light distribution (in $1/{\mathrm{mm}}^{2}$) across source–detector arrays within a mesh-based medium.

**Fig. 2:**
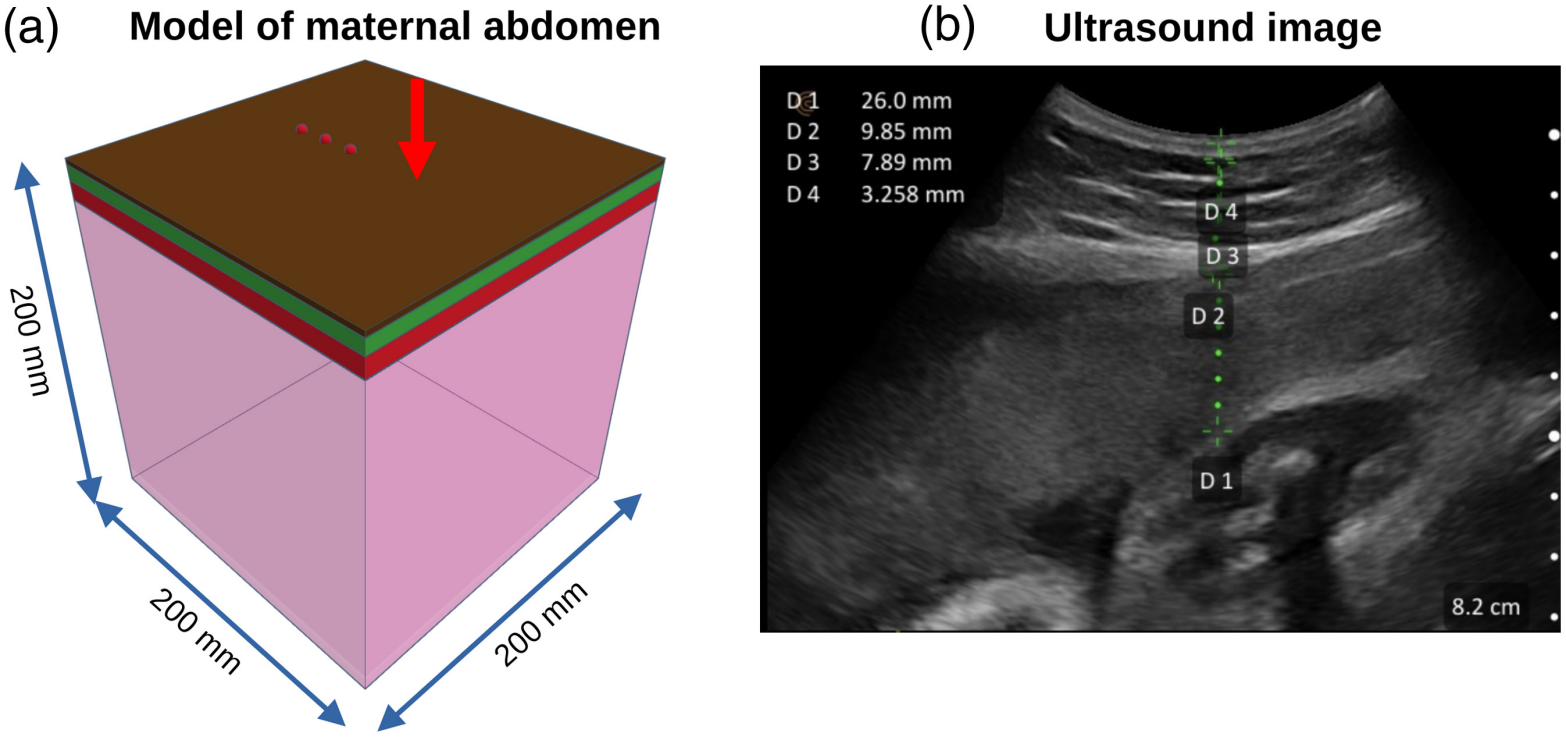
Developing a model of maternal abdominal structures. (a) Model of a maternal abdomen and acquisition setup using the adjustable distance broadband NIRS device Mini CYRIL.^26^ The dark green, light green, purple, and blue layers represent the skin, adipose tissue, muscle, and placenta layers, respectively. The light source is depicted as a red arrow, and the three different positions of the fiber detector of the adjustable-distance CW-NIRS device are shown as red disks (source–detector separations of 30, 40, and 50 mm). (b) Ultrasound image used for measuring layer thickness (D1: placenta thickness, D2: muscle thickness, D3: adipose tissue thickness, D4: skin thickness). In the rest of the paper, the term placenta depth refers to the distance from skin to placenta: D2+D3+D4

In our study, we modeled the light propagation in the maternal abdomen at 780 nm. We choose this wavelength because it is usually included in NIRS devices and the optical properties of the solid phantom used in the calibration procedure are known for this wavelength, see Sec. 2.5. The mesh-based medium dimensions were 200×200×200  mm. The maternal abdomen was modeled as a four-layer volume (skin, adipose tissue, muscle, and placenta), see Fig. 2. In this model, the muscle is a composite of both rectus abdominis and myometrium (uterus). For each layer, the mesh medium was composed of tetrahedral elements having known optical properties. In this study, we fixed the maximum tetrahedral element volume at $0.5\;{\mathrm{mm}}^{3}$ for skin, $0.5\;{\mathrm{mm}}^{3}$ for adipose tissue, $1\;{\mathrm{mm}}^{3}$ for muscle, and $100\;{\mathrm{mm}}^{3}$ for placenta. In our simulations with Redbird, we applied the partial-current boundary condition to account for photon losses at the tissue–air interface.^33^

A point light source was positioned perpendicular to the surface of the volume, along with three point detectors placed at distances of 30 to 50 mm from the source. Using Redbird, it is possible to calculate the fluence distribution (in $1/{\mathrm{mm}}^{2}$ considering a unitary light source) across the mesh-based volume. A total of 51,840 simulations were performed with varying parameters:

• melanin volume fraction (in %) ∈[2.55,15.5,30.5] that was estimated from the Fitzpatrick scale^34^
• skin thickness (in mm) ∈[1,5] in steps of 1 mm
• adipose tissue thickness (in mm) ∈[1,2,4,8,16]
• muscle thickness (in mm) ∈[3,7,10,13,20,25]
• muscle oxygen saturation (in %) ∈[40,60,80]
• placenta oxygen saturation (in %) ∈[40,60,80]
• blood volume in the muscle layer (in Mol) ∈[15,25,35,50]
• blood volume in the placenta layer (in Mol) ∈[15,25,35,50].

After the simulations, fluence values were obtained at each node of the tetrahedral mesh. The fluence distribution was then interpolated onto a 200×200×200  mm volume with $1\;{\mathrm{mm}}^{3}$ resolution. The diffuse reflectance ϕ(x,y) (in ${\mathrm{mm}}^{-2}$) located at the surface (x,y) of the tissue represents the quantity of light exiting the tissue. It can be defined as the integral of the radiance over the tissue backward hemisphere. The radiance is expressed as the sum of two terms proportional to the fluence and to the flux^35^:

$$\phi (x,y)=\int_{\theta =0}^{\pi /2}\int_{\varphi =0}^{2\pi }[1-R_{\mathrm{fres}}(\theta )]\times \frac{1}{4\pi }[F(x,y,z=0)+3D\frac{\partial F(x,y,z=0)}{\partial dz}\;\cos(\theta )]\cos(\theta )\sin(\theta )d\theta \;d\varphi ,$$ (1)

where $R_{\mathrm{fres}}(\theta )$ is the Fresnel reflection coefficient (in arbitrary units) for a photon with an incident angle θ (in radians) relative to the normal to the boundary. F(x,y,z=0) is the fluence calculated at voxel location (x,y,z=0), $\frac{\partial F(x,y,z=0)}{\partial dz}$ is the flux measured at the voxel location (x,y,z=0), $D=\frac{1}{3(\mu_{a}+\mu_{s}^{\prime })}$ (in mm) is the diffusion constant, $\mu_{a}$ and $\mu_{s}^{\prime }$ are the absorption and reduced scattering coefficients of the top layer (in ${\mathrm{mm}}^{-1}$).

Diffuse reflectance values were summed over a disk area with a 2.3 mm diameter to model the detector fiber of the Mini-CYRIL device. Diffuse reflectance at the detector level was interpolated on a $0.1\;{\mathrm{mm}}^{2}$ grid. The detector fiber diameter can be directly adjusted in our pipeline to simulate other CW-NIRS devices.

### 2.2 Optical Properties of the Maternal Abdomen

The modeled tissue included information about its optical properties: absorption coefficient ($\mu_{a}$ in ${\mathrm{mm}}^{-1}$), reduced scattering coefficient ($\mu_{s}^{\prime }$ in ${\mathrm{mm}}^{-1}$), anisotropy coefficient (g), and refractive index (n). The anisotropy coefficients, refractive indices, the reduced scattering coefficients, and the parameters used to calculate the absorption coefficients of the tissues are listed in Table 1. Absorption and scattering coefficients of skin, adipose tissue, muscle, and placenta layers used in the study have been reported in Table S3 in the Supplementary Material.

**Table 1:** Optical parameters and chemical composition of the modeled tissue. n represents the refractive index, g is the anisotropy coefficient, $\mu_{s}^{\prime }(\lambda )$ (in ${\mathrm{mm}}^{-1}$) is the wavelength-dependent reduced scattering coefficient, $F_{W}$ is the volume fraction of water (in %), $F_{F}$ is the volume fraction of fat (in %), $C_{\mathrm{HbT}}$ is the molar concentration of total haemoglobin, and ${\mathrm{SatO}}_{2}$ is the oxygen saturation of the tissue (in %).

	Skin	Adipose tissue	Muscle (rectus abdominis and uterus)	Placenta
n	1.47^34^	1.37^36^	1.37^36^	1.4^37^
g	0.83^34^	0.9^36^	0.9^36^	0.9^37^
$\mu_{s}^{\prime }(\lambda )$ (in ${\mathrm{mm}}^{-1}$)	$4.6(\frac{\lambda }{500}{)}^{-1.421}$ ^36^	$1.84(\frac{\lambda }{500}{)}^{-0.672}$ ^36^	$1.3(\frac{\lambda }{500}{)}^{-0.926}$ ^36^	$1.6619(\frac{\lambda }{500}{)}^{-1.426}$ ^16^
$F_{W}$ (in %)	0	80	76	85
$F_{F}$ (in %)	0	20	0	0
$C_{\mathrm{HbT}}$ (in μMol)	4.7 (dermis)^38^	0	[15;25;35;50]	[15;25;35;50]
${\mathrm{SatO}}_{2}$ (in %)	39 (dermis)^36^	—	[40;60;80]	[40;60;80]

The wavelength-dependent absorption coefficients $\mu_{a}(\lambda )$ (in ${\mathrm{mm}}^{-1}$) for the adipose tissue, muscle, and placenta layers were derived from the chemical composition of the tissues^36^:

$$\mu_{a}(\lambda )=F_{F}.\mu_{a_{F}}(\lambda )+F_{W}.\mu_{a_{W}}(\lambda )+\log(10).C_{\mathrm{HbT}}.({\mathrm{SatO}}_{2}.\epsilon_{{\mathrm{HbO}}_{2}}(\lambda )+(1-{\mathrm{SatO}}_{2}).\epsilon_{\mathrm{Hb}}(\lambda )),$$ (2)

with $F_{W}$ is the volume fraction of water (in %), $F_{F}$ the volume fraction of fat (in %), $C_{\mathrm{HbT}}$ the molar concentration of total haemoglobin (in M), ${\mathrm{SatO}}_{2}$ the oxygen saturation of the tissue (in %), $\mu_{a_{F}}$ the absorption coefficient of fat (in ${\mathrm{mm}}^{-1}$), $\mu_{a_{W}}$ the absorption coefficient of water (in ${\mathrm{mm}}^{-1}$), and $\epsilon_{{\mathrm{HbO}}_{2}}$ and $\epsilon_{\mathrm{Hb}}$ the molar extinction coefficient of oxy- and deoxygenated hemoglobin, respectively (in ${\mathrm{Mol}}^{-1}.{\mathrm{mm}}^{-1}$).

The human skin consists of different layers: the epidermis and the dermis. The baseline absorption of both epidermis and dermis (in ${\mathrm{mm}}^{-1}$) is approximated by the following expression:^39^

$$\mu_{a_{\text{Skin}-\text{Baseline}}}(\lambda )=7.84.10^{7}\lambda^{-3.255}.$$ (3)

The absorption of epidermis (in ${\mathrm{mm}}^{-1}$) is usually dominated by melanin absorption, which is defined as:^36^

$$\mu_{a_{\mathrm{mel}}}=51.9(\frac{\lambda }{500}{)}^{-3}.$$ (4)

The net epidermal absorption coefficient, $\mu_{a_{\mathrm{epi}}}$ (in ${\mathrm{mm}}^{-1}$), combines the baseline skin absorption and the melanin absorption:

$$\mu_{a_{\mathrm{epi}}}=f_{\mathrm{mel}}.\mu_{a_{\mathrm{mel}}}+(1-f_{\mathrm{mel}}).\mu_{a_{\text{Skin}-\text{Baseline}}},$$ (5)

with $f_{\mathrm{mel}}$ the volume fraction of melanin in the epidermis (in %). This value changes as a function of the Fitzpatrick scale, which is an indicator of the skin tone:^34^ $f_{\mathrm{mel}}\in [2.55\%;30.5\%]$. The total optical absorption coefficient of the dermis depends on a minor baseline skin absorption and a dominant hemoglobin absorption due to the cutaneous blood perfusion:^40^

$$\mu_{a_{\text{derm}}}=(1-F_{B}).\mu_{a_{\text{Skin}-\text{Baseline}}}+\log(10).C_{\mathrm{HbT}}.({\mathrm{SatO}}_{2}.\epsilon_{{\mathrm{HbO}}_{2}}(\lambda )+(1-{\mathrm{SatO}}_{2}).\epsilon_{\mathrm{Hb}}(\lambda )),$$ (6)

with $\mu_{W}$ the absorption coefficient of water (in ${\mathrm{mm}}^{-1}$), $C_{\mathrm{HbT}}$ the molar concentration of total hemoglobin (in Mol), ${\mathrm{SatO}}_{2}$ the oxygen saturation of the tissue (in %), and $\epsilon_{{\mathrm{HbO}}_{2}}$ and $\epsilon_{\mathrm{Hb}}$ the molar extinction coefficient of oxy- and deoxygenated hemoglobin, respectively (in ${\mathrm{Mol}}^{-1}.{\mathrm{mm}}^{-1}$). $F_{B}=0.20\%$^38^ is the volume fraction of blood (in %) in dermis. In our study, we modeled the skin as a single layer. Thus, the volume fraction of epidermis $F_{\mathrm{epi}}$ and epidermis $F_{\text{derm}}$ has to be taken into consideration to calculate the absorption coefficient of the skin layer:

$$\mu_{a_{\text{skin}}}=F_{\mathrm{epi}}.\mu_{a_{\mathrm{epi}}}+F_{\text{derm}}.\mu_{a_{\text{derm}}}.$$ (7)

$F_{\mathrm{epi}}=2.72\%$ and $F_{\text{derm}}=97.28\%$ have been extracted from Ref. 41, in which the authors performed measurements of epidermis and dermis thickness for the abdomen.

### 2.3 Clinical Measurements

Measurements of skin tones and thickness of the skin, adipose tissue, and muscle layers were performed on 142 healthy participants during pregnancy at the Institute for Women’s Health at University College London Hospital (UK). The measurements were approved by the local ethics committee of University College London Hospital, and the participating patients provided written consent. The thickness values were obtained from manual segmentation of the ultrasound images, performed by an expert immediately after acquisition. The error in thickness values is related to the spatial resolution of the ultrasound probe (0.5 mm). The ultrasound probe was carefully placed on the maternal abdomen to minimize the distance from the skin surface to the placenta. For this reason, measurements were not collected from the same location for all participants. In this study, participants had anterior or posterior placentas. For the mothers having posterior placentas that are close to the spine, the clinical team asked the participants to lie on their sides to minimize the distance from the skin surface to the placenta.

**Fig. 3:**
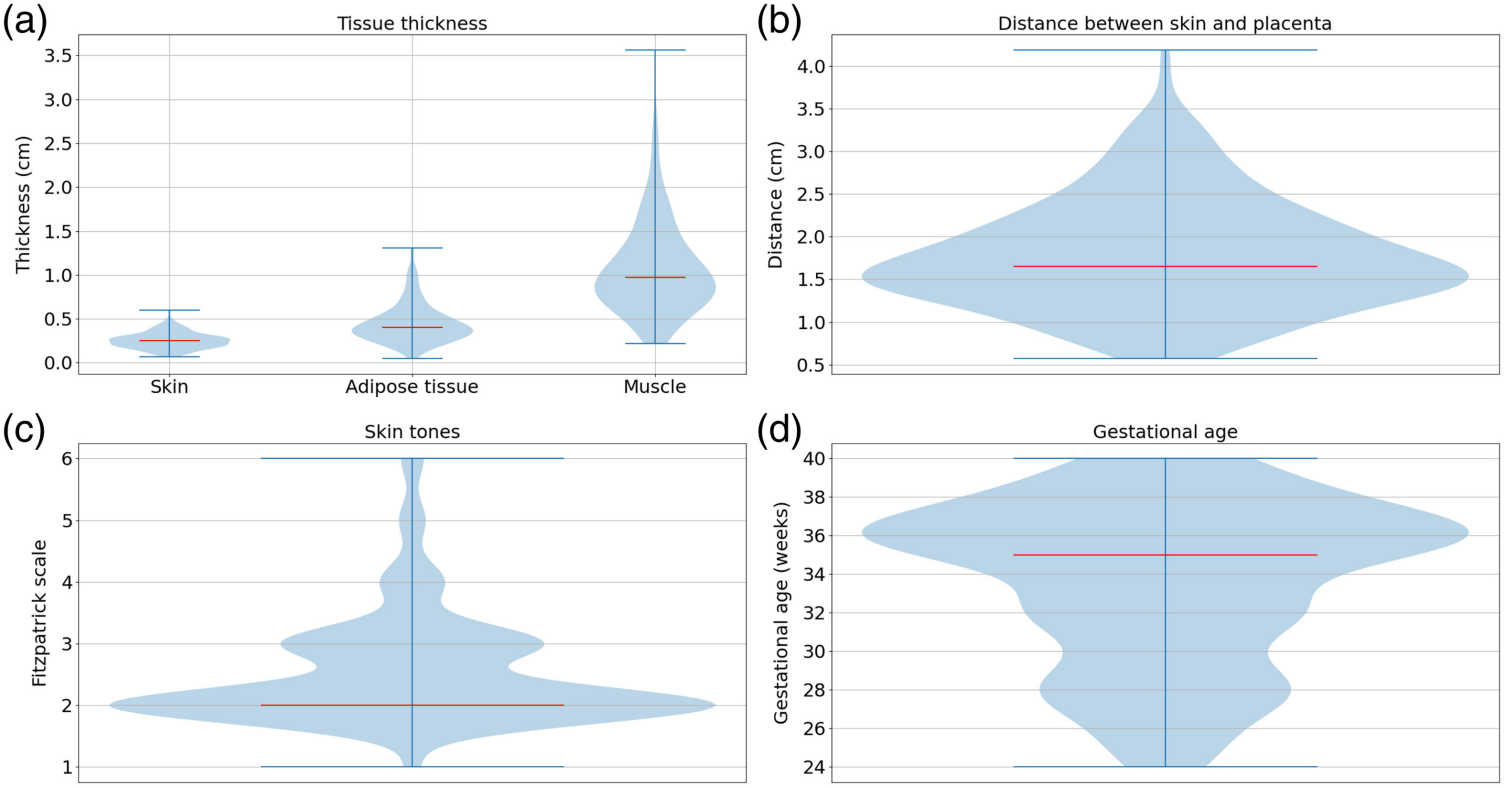
Information on the abdominal morphology, the skin tones, and the gestational age of the 142 healthy participants included in this study. (a) Tissue thickness. (b) Distance between skin and placenta. (c) Fitzpatrick scale. (d) Gestational ages. The median values are represented by red lines.

The skin tone was evaluated with the Fitzpatrick scale (see Fig. 3), which is a subjective assessment of skin tone based on visual inspection.^42^ The Fitzpatrick scale was directly evaluated on the patient’s abdomen, in real time, during the appointment using a scale displayed on a screen. The evaluation of the Fitzpatrick scale is subjective but is the most clinically validated tool. The scale can be easily evaluated during the appointment sessions and did not require any further instruments. In this study, we converted the Fitzpatrick scale into melanin volume fraction using the lookup table provided by Karsten and Smit.^34^ Darker skin tones are associated with higher melanin volume fractions because melanin is the primary pigment responsible for skin coloration and its concentration increases with pigmentation.

### 2.4 Placenta Sensitivity

We used the simulated fluence volumes and the adjoint method^43^ to calculate sensitivity matrices. By normalizing the sensitivity matrix by its sum, we obtained the sensitivity probability volume P (in %) of photon propagation between a pair of point-source and point-detector.^44^ In the rest of the paper, the term placenta sensitivity $S_{\text{Placenta}}$ (in %) refers to the probability that emitted light passes through the placenta for a given source–detector pair. However, it does not reflect the probability that the emitted light is detected at detector D with a signal level significantly above the noise. In other words, emitted light may reach the placenta, increasing placenta sensitivity, but in some cases, the signal cannot be detected due to high tissue absorption and noise. Placenta sensitivity is calculated by summing the sensitivity probability volume P on the slice related to the placenta layer:

$$S_{\text{Placenta}}=\underset{x}{\sum }\underset{y}{\sum }\overset{z_{N_{2}}}{\underset{z=z_{N_{1}}}{\sum }}P(x,y,z),$$ (8)

with $z_{N_{1}}$ and $z_{N_{2}}$ the depth indexes of the placenta layer. The placenta sensitivity refers to the capability of a perfect device (infinite SNR) to scan the placenta; however, it does not include the detection probability of a measurement to be above the noise level of the detector D.

### 2.5 Calibration of Simulated Data

**Fig. 4:**
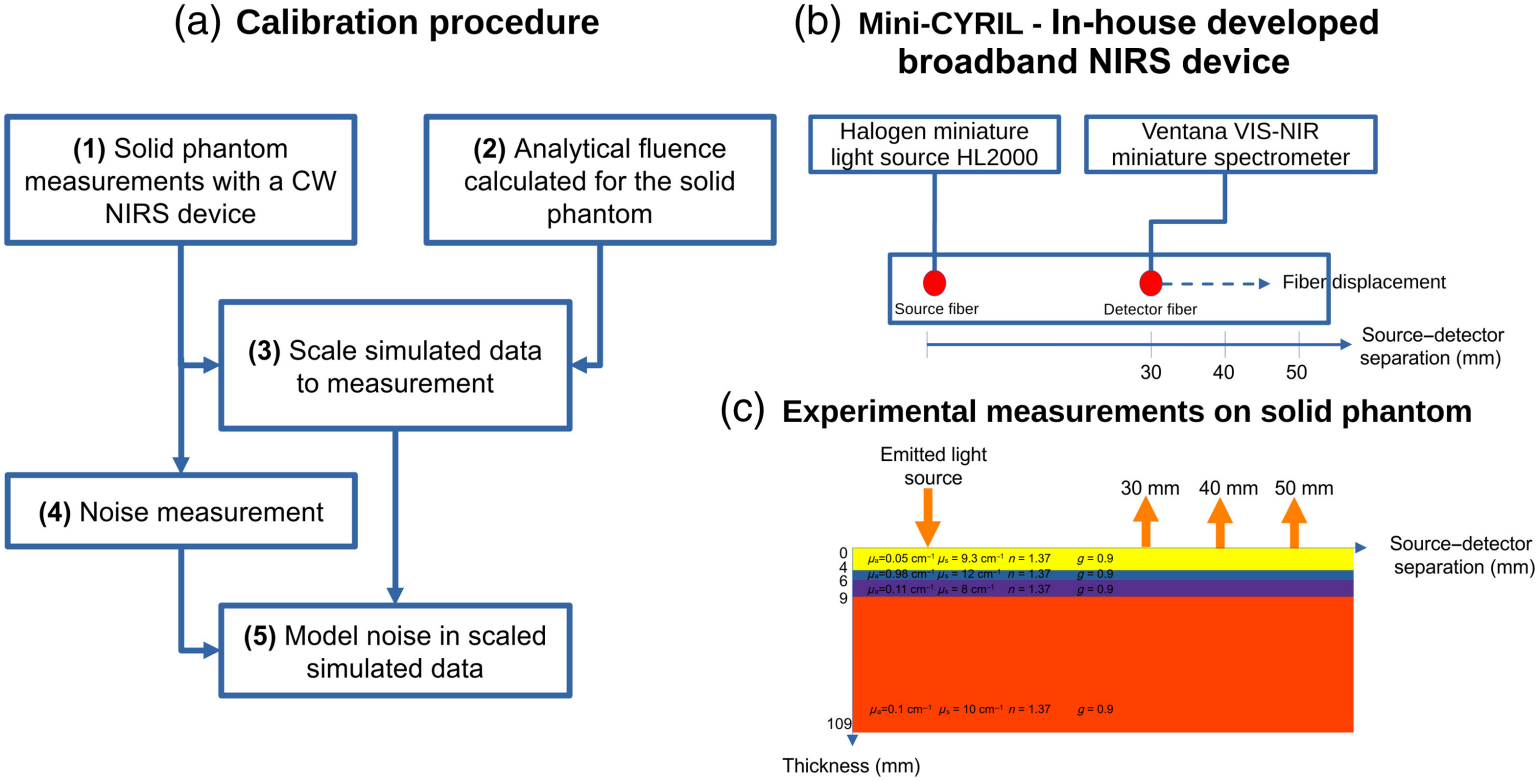
Flowchart for the calibration procedure of simulated data to scale CW-NIRS measurements. (a) Calibration procedure. (b) Schematic of the in-house developed broadband CW-NIRS device. (c) Schematic of the solid phantom used in this study.

Simulated data obtained with Redbird software did not take into account factors associated with each source and detector. These factors include detector sensitivities, fiber coupling efficiency, optical fiber light leakage, bending, and fiber-tissue coupling (e.g., hair and pressure differences). To mimic real CW-NIRS measurements, which take into account system-dependent factors, we used a calibration procedure (see Fig. 4). With this procedure, simulated data of a solid phantom were scaled to match the experimental data measured for a solid phantom. The scaling factors are then used to calibrate simulated data of the maternal abdomen. Finally, noise was added to the calibrated simulated data to model realistic measurements.

In NIRS studies, it is common to use solid phantoms to calibrate the measurement system against simulated data. This calibration is a necessary step to enable accurate estimation of optical properties via inverse problem solutions.^45^^–^^47^ In this study, simulated diffuse reflectance values were calibrated against measurements from an in-house developed adjustable distance broadband CW-NIRS device^26^ to replicate CW-NIRS measurements of a maternal abdomen. However, the calibration procedure remains identical when applied to any other CW-NIRS device.

The calibration procedure and the calculation of the detection probability consisted of five steps:

1. We performed diffuse reflectance measurements $\phi_{\mathrm{Mes}}^{\text{Phantom}}$ at 780 nm of a multilayered phantom with an adjustable distance broadband NIRS device, see Fig. 4 and Table 2.

**Table 2:** Acquisition details of the adjustable distance NIRS device Mini CYRIL.^26^

	Mini CYRIL^26^
Source–detector separations (mm)	30, 40, 50
Integration time (s)	1, 5, 10
Acquisition duration (min)	8, 10, 20

The Mini-CYRIL is an in-house developed adjustable distance broadband CW-NIRS device. It uses a thermally stabilized miniature white-light source (HL-2000-HP)—a 20 W tungsten halogen lamp (Ocean Optics, Orlando, Florida, United States). The source and detector optical fibers are identical bundles and custom-built by Loptek (Germany) for maximum illumination and light collection. A detector fiber was connected to a Ventana VIS-NIR miniature spectrometer (Ocean Optics). The device is totally customizable: we performed successive acquisitions with three different source–detector separations (with displacement of the detection fiber) and three different values of the spectrometer integration time, see Table 2.

The multilayered phantom (BioPixS Limited, Cork, Ireland) was composed of four solid layers having known optical properties similar to those of biological tissue, see Table 3. These optical properties do not correspond to those of a maternal abdomen. However, the multilayer structure of the phantom is designed to mimic a calibrated maternal abdomen.

**Table 3:** Optical properties at 780 nm and thickness of the multilayer phantom.

	Absorption coefficient (${\mathrm{mm}}^{-1}$)	Scattering coefficient (${\mathrm{mm}}^{-1}$)	Thickness (mm)
First layer	0.005	0.93	4
Second layer	0.098	1.2	2
Third layer	0.011	0.8	3
Fourth layer	0.01	1	100

For the four layers, the values of the anisotropy coefficients and of the refractive indexes were 0.9 and 1.37, respectively. Measurements of the solid phantom have been performed at 780 nm because the optical properties of the multilayer phantom were only known for this wavelength.

2. We calculated the diffuse reflectance values $\phi_{\mathrm{Simu}}^{\text{Phantom}}$ (in arbitrary units) of the multilayered phantom at the level of the detectors with the Redbird software.^29^^,^^30^ These diffuse reflectance values were calculated at 780 nm for each detector.
3. For each detector D, a scaling coefficient $C_{\text{scale}}$ was calculated to scale $\phi_{\mathrm{Simu}}^{\text{Phantom}}$ to $\phi_{\mathrm{Mes}}^{\text{Phantom}}$ such that
  $$C_{\text{scale}}(D)=\frac{\phi_{\mathrm{Mes}}^{\text{Phantom}}(D)}{\phi_{\mathrm{Simu}}^{\text{Phantom}}(D)}.$$ (9)
  $C_{\mathrm{scale}}(D)$, $\phi_{\mathrm{Mes}}^{\mathrm{Phantom}}(D)$ and $\phi_{\mathrm{Simu}}^{\mathrm{Phantom}}(D)$ are expressed in arbitrary units.
4. For each detector, we calculated the noise level in the experimental signal measured with the CW-NIRS device (standard deviation of $\phi_{\mathrm{Mes}}^{\text{Phantom}}$). This noise level corresponded to the minimum detectable diffuse reflectance value $\phi_{\min}$ (in arbitrary units).
5. For each detector D, simulated data of the maternal abdomen (see Sec. 2.1) were calibrated, and sensor noise was added to the scaled data
  $$\phi_{\mathrm{Simu},\mathrm{calib}}^{\text{Placenta}}(D)=C_{\text{scale}}(D).\phi_{\mathrm{Simu}}^{\text{Placenta}}(D)+n,$$ (10)
  with n (in arbitrary units), the sensor noise modeled as a vector of 100,000 elements calculated with the distribution $N(0,\phi_{\min}(D))$.^48^

### 2.6 Detection and Scanning Probability of CW-NIRS Device

The detection probability represents the probability of a detector acquiring a signal that is significantly above the noise level. In this study, this probability was calculated with the Mini-CYRIL device, but it can be applied to any CW-NIRS device using the calibration procedure. The detection probability was obtained with a one-sided T-test using the following hypotheses:

$$\left\{ \begin{array}{c} H_{0}:\;\phi_{\mathrm{Simu},\mathrm{calib}}^{\text{Placenta}}(D)=\phi_{\min}(D)\\ H_{1}:\;\phi_{\mathrm{Simu},\mathrm{calib}}^{\text{Placenta}}(D)>\phi_{\min}(D) \end{array}.\right.$$ (11)

Detection probability values were directly derived from the one-sided T-test $p_{\text{value}}$, using the relation $100.(1-p_{\text{value}})$. We defined the placental scanning probability as the multiplication of the detection probability by the placenta sensitivity, see Sec. 2.4 (independent probabilities). It represents the probability that detector D receives the minimum detectable irradiation and that the placenta is scanned for the given source–detector separation.

### 2.7 Placental Tissue Oxygenation

To evaluate the accuracy of adjustable distance CW-NIRS devices for measuring placental tissue oxygenation ${\mathrm{PltO}}_{2}$ (in %), we implemented the SRS algorithm.^22^ This technique has been used in several clinical studies using CW-NIRS to detect at-risk pregnancies,^17^^,^^23^^–^^25^ but the accuracy of this model for measuring placental tissue oxygenation has not been studied. By measuring light attenuation at multiple source–detector separations, SRS analyzes how the attenuation changes with distance. SRS uses a multidistance measurement of attenuation A to calculate its slope against detector distance ρ. Based on the zero-boundary condition solution to the diffusion equation in a semi-infinite medium, the slope of attenuation versus distance ∂A/∂ρ can be used to estimate a scaled absorption coefficient $k.\mu_{a}$ (in ${\mathrm{mm}}^{-1}$), with k an unknown constant. As the tissue absorption is mainly due to oxy- and deoxygenated hemoglobin, scaled concentration of oxy- and deoxygenated hemoglobin ($k.C_{{\mathrm{HbO}}_{2}}$ and $k.C_{\mathrm{Hb}}$ in Mol) can be derived from $k.\mu_{a}$ using the modified Beer–Lambert law. Finally, the placental tissue oxygen saturation ${\mathrm{PltO}}_{2}$ (in %) can be calculated

$${\mathrm{PltO}}_{2}=\frac{k.C_{{\mathrm{HbO}}_{2}}}{k.C_{{\mathrm{HbO}}_{2}}+k.C_{\mathrm{Hb}}}.$$ (12)

All mathematical details of the SRS algorithm can be found in Scholkmann et al.^49^

In our study, we simulated diffuse reflectance values at three source–detector separations (i.e., 30, 40, and 50 mm) and six wavelengths (i.e., 780, 810, 830, 840, 850, and 890 nm) for three participants with fixed tissue layer thicknesses (placenta depths of 10, 16, and 20 mm). For the three participants, the thicknesses for each layer corresponded to the 25th, 50th, and 75th percentiles of the thickness distribution calculated on the 142 healthy participants, see Fig. 3. For each participant, several parameters vary: melanin volume fractions (2.55%, 15.5%, and 30.5%), oxygen saturation of the muscle and placenta layers. Blood volumes of the muscle and placenta layers were fixed to 35  μM. These values fall within the range of variations reported for muscle^50^ and placenta.^16^ The SRS algorithm was applied to these simulated data to evaluate the accuracy of adjustable distance CW-NIRS devices for measuring placental tissue oxygenation.

## 3 Results

### 3.1 Phantom Calibration

**Fig. 5:**
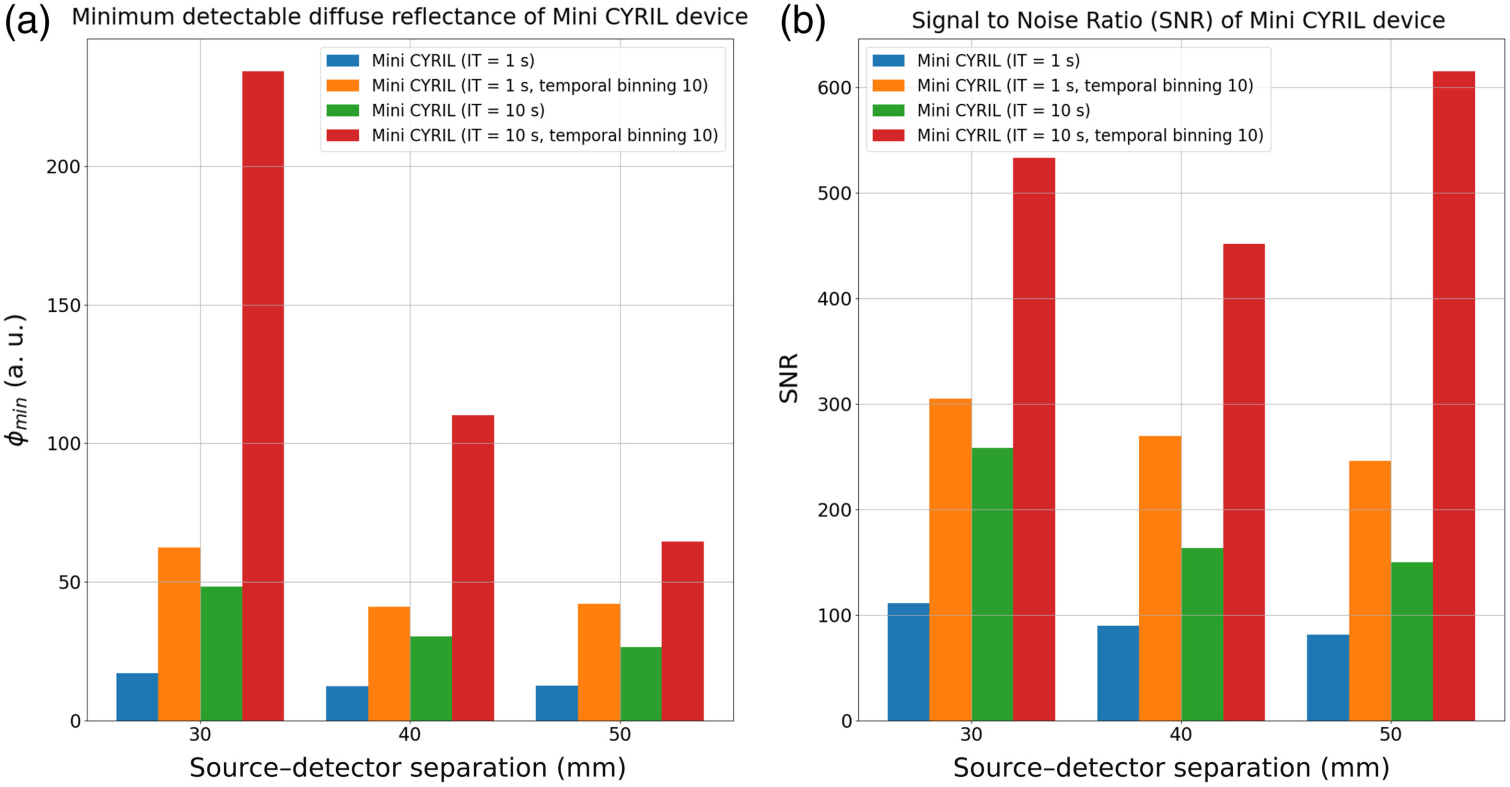
Phantom calibration using Mini CYRIL. Diffuse reflectance data of the solid phantom (see Fig. 4) have been collected with the Mini-CYRIL device for 30, 40, and 50 mm source–detector separations and with integration times of 1 and 10 s. A temporal binning of 10 points was applied to the data after acquisition to improve the SNR. (a) Minimum detectable diffuse reflectance. (b) SNR of the signal collected. IT stands for integration time.

In Fig. 5, we represented the minimum detectable diffuse reflectance values calculated with the calibration procedure (see Sec. 2.5) and the SNR of the signals measured on the multilayered solid phantom with the Mini CYRIL device with integration times of 1 and 10 s. Data were calculated with and without a temporal binning. A temporal binning of 10 means that consecutive groups of 10 diffuse reflectance data points were summed to mimic the multiplication of the integration time by a factor of 10.

In graph (A), the minimum detectable diffuse reflectance corresponds to the standard deviation of the experimental signal measured in the solid phantom with the CW-NIRS device. Graph (B) shows the SNR of the experimental signal measured under the same conditions. Without temporal binning, the SNR decreased with increasing source–detector separation and increased with longer integration times. The minimum detectable diffuse reflectance decreased with source–detector separation, as the standard deviation of the measurements also decreased, see Fig. S1 in the Supplementary Material. This is consistent with the decrease in SNR at larger separations. The minimum detectable diffuse reflectance also increased with temporal binning as the binning procedure was defined as the sum of 10 consecutive points.

The SNR measured at a 50 mm source–detector separation, for an integration time of 10 s and a binning of 10, is higher than that at 40 mm source–detector separation. This indicates that the SNR calculation is not reliable at 50 mm source–detector separation. When multiple exposures are binned, both the signal and the photon noise increase. If the photon signal is close to or below the background noise level, the SNR becomes unreliable.

### 3.2 Detection Probability

**Fig. 6:**
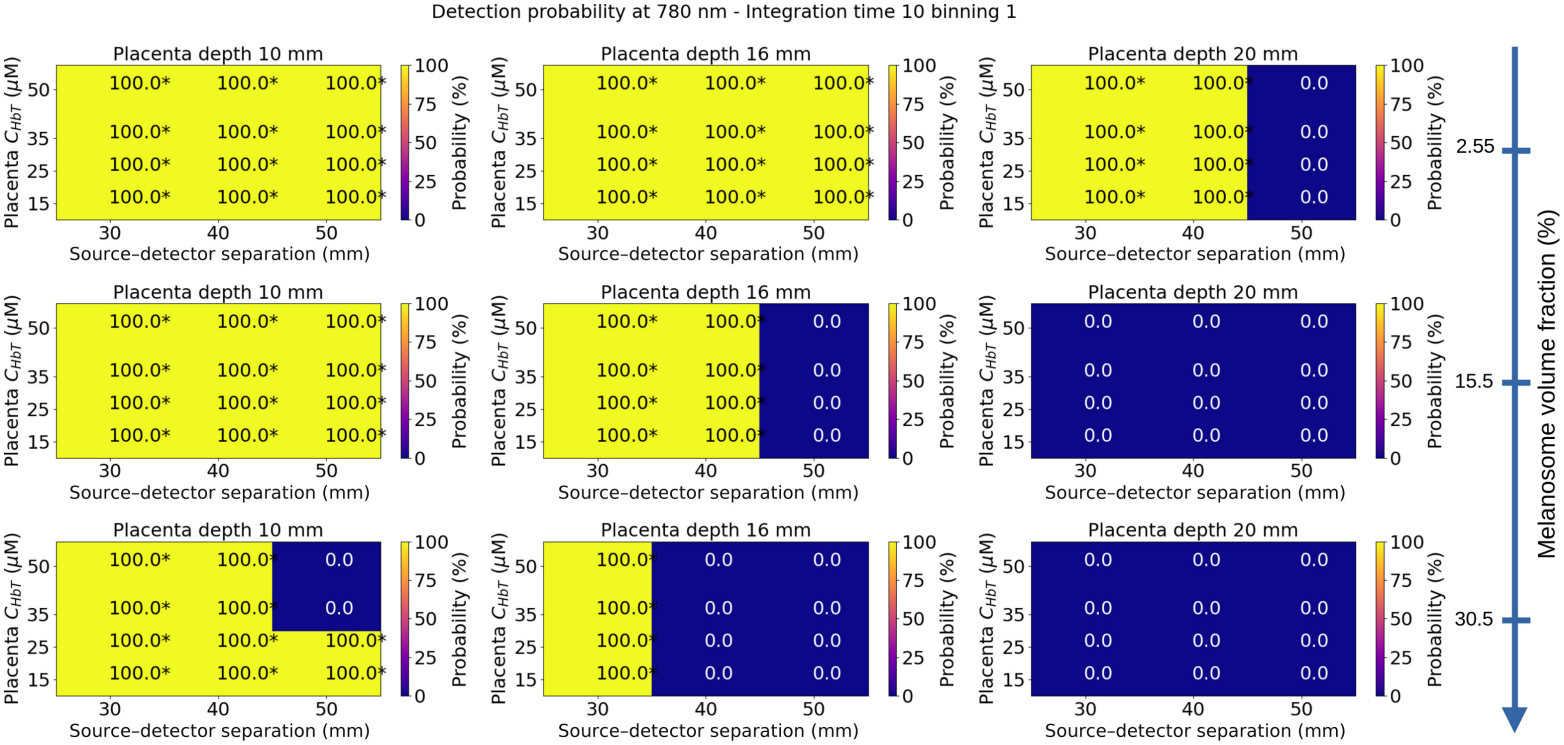
Detection probability of the Mini CYRIL at 780 nm for an exposure time of 10 s and no temporal binning as a function of the placenta blood volume, source–detector separation, melanosome volume fraction and placenta depth. NIRS signals were simulated for these values; the other parameters were fixed (muscle blood volume: 25  μMol, muscle ${\mathrm{SatO}}_{2}=60\%$, placenta ${\mathrm{SatO}}_{2}=80\%$). Absorption and scattering coefficients of the simulated maternal abdomen are listed below: $\mu_{a}^{\text{skin}}=0.040$, 0.088, and $0.144\;{\mathrm{mm}}^{-1}$ for a melanosome volume fraction of 2.55%, 15.5%, and 30.5%, respectively. $\mu_{s}^{\text{skin}}=14.38\;{\mathrm{mm}}^{-1}$, $\mu_{a}^{\text{Adipose tissue}}=0.002\;{\mathrm{mm}}^{-1}$, $\mu_{s}^{\text{Adipose tissue}}=13.64\;{\mathrm{mm}}^{-1}$, $\mu_{a}^{\text{Muscle}}=0.0069\;{\mathrm{mm}}^{-1}$, $\mu_{s}^{\text{Muscle}}=8.61\;{\mathrm{mm}}^{-1}$, $\mu_{a}^{\text{Placenta}}=0.0049$, 0.0067, 0.0085, and $0.0113\;{\mathrm{mm}}^{-1}$ for $C_{\mathrm{HbT}}^{\text{Placenta}}=15$, 25, 35, and 50  μM, respectively. $\mu_{s}^{\text{Placenta}}=8.81\;{\mathrm{mm}}^{-1}$. For each placenta depth, the thickness of each tissue layer is indicated in Table 4. Detection probability values were directly derived from the one-sided T-test $p_{\text{value}}$, using the relation $100.(1-p_{\text{value}})$. Values marked with stars * indicated that the null hypothesis has been rejected at the 5% significant level (detection probability >95%), see Eq. (11).

**Fig. 7:**
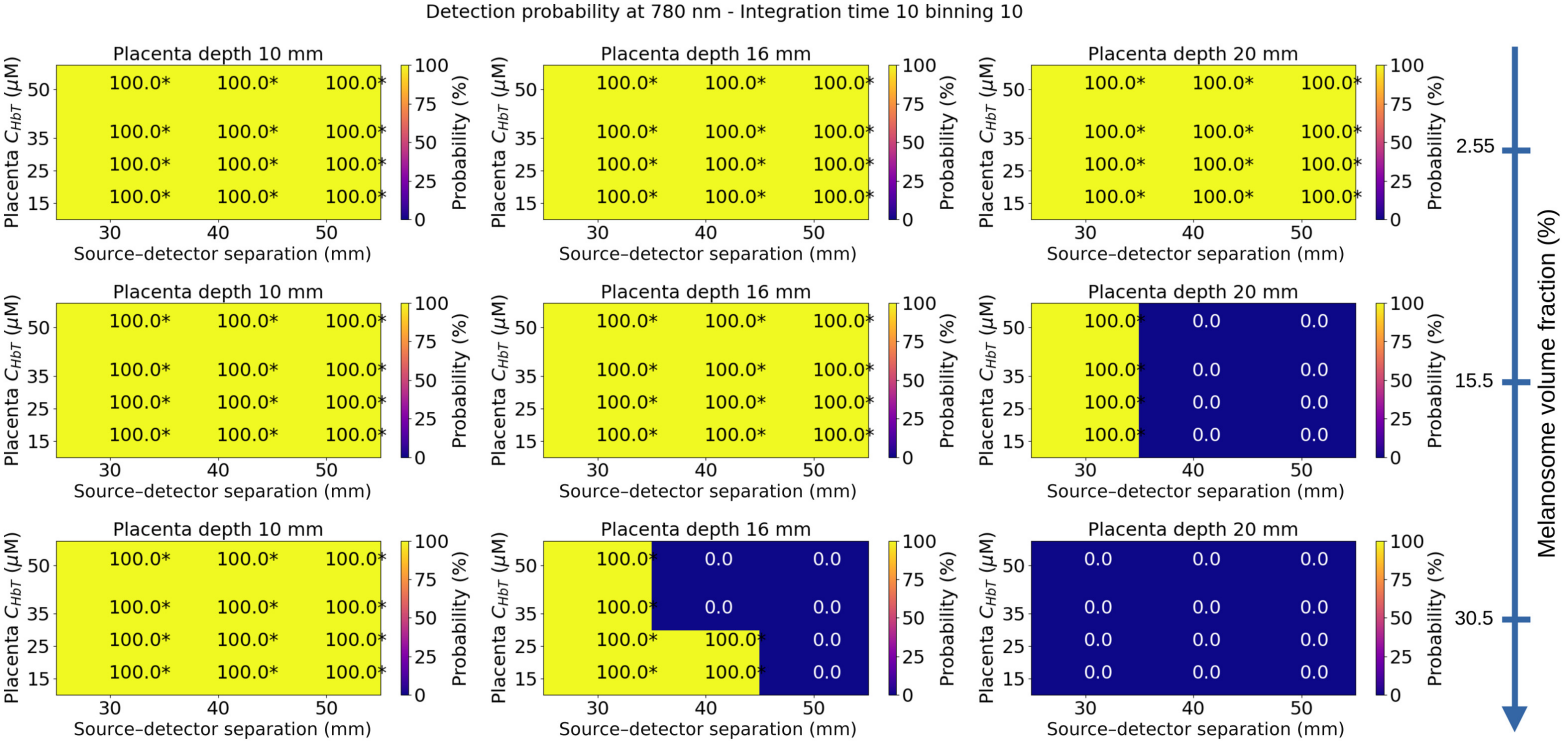
Detection probability of the Mini CYRIL at 780 nm for an exposure time of 10 s and a temporal binning of 10 points as a function of the placenta blood volume, source–detector separation, melanosome volume fraction, and placenta depth. NIRS signals were simulated for these values, the other parameters were fixed (muscle blood volume: 25  μMol, muscle ${\mathrm{SatO}}_{2}=60\%$, placenta ${\mathrm{SatO}}_{2}=80\%$). Absorption and scattering coefficients of the simulated maternal abdomen are listed below: $\mu_{a}^{\text{skin}}=0.040$, 0.088, and $0.144\;{\mathrm{mm}}^{-1}$ for a melanosome volume fraction of 2.55%, 15.5%, and 30.5%, respectively. $\mu_{s}^{\text{skin}}=14.38\;{\mathrm{mm}}^{-1}$, $\mu_{a}^{\text{Adipose tissue}}=0.002\;{\mathrm{mm}}^{-1}$, $\mu_{s}^{\text{Adipose tissue}}=13.64\;{\mathrm{mm}}^{-1}$, $\mu_{a}^{\text{Muscle}}=0.0069\;{\mathrm{mm}}^{-1}$, $\mu_{s}^{\text{Muscle}}=8.61\;{\mathrm{mm}}^{-1}$, $\mu_{a}^{\text{Placenta}}=0.0049$, 0.0067, 0.0085, and $0.0113\;{\mathrm{mm}}^{-1}$ for $C_{\mathrm{HbT}}^{\mathrm{Placenta}}=15$, 25, 35, and 50  μM, respectively. $\mu_{s}^{\text{Placenta}}=8.81\;{\mathrm{mm}}^{-1}$. For each placenta depth, the thickness of each tissue layer is indicated in Table 4. Detection probability values were directly derived from the one-sided T-test $p_{\text{value}}$, using the relation $100.(1-p_{\text{value}})$. Values marked with stars * indicated that the null hypothesis has been rejected at the 5% significant level (detection probability >95%), see Eq. (11).

In Figs. 6 and 7, we represented the detection probabilities of Mini CYRIL using two different hardware configurations (1 to 780 nm, 10 s exposure time, and no temporal binning; 2 to 780 nm, 10 s exposure time, and 10 points temporal binning). This configuration was selected because it provides improved SNR, see Fig. 5. The detection probabilities of the Mini CYRIL at 780 nm calculated for other configurations (integration times 1 and 5 s) are represented in Figs. S1–S4 in the Supplementary Material. The probabilities were plotted as a function of the placenta blood volume, source–detector separation, melanosome volume fraction, and placenta depth. The thickness of the layers of the maternal abdomen model used for the calculation of the detection probabilities is shown in Table 4. The reported thicknesses correspond to the 25th, 50th, and 75th percentiles of measurements derived from ultrasound images, see Fig. 3.

**Table 4:** Layers thickness used for the calculation of the detection probability, placenta sensitivity, and placental tissue oxygenation in Secs. 3.2, 3.3, and 3.5, respectively.

Thickness percentiles (%)	Distance from skin to placenta (placenta depth in mm)	Skin thickness (mm)	Adipose tissue thickness (mm)	Muscle thickness (mm)
25	10	1	2	7
50	16	2	4	10
75	20	3	5	12

In Fig. 6, the detection probabilities of the Mini CYRIL at 780 nm were calculated with an integration time of 10 s and no temporal binning. With this hardware configuration, a sufficient quantity of photons is detected for 30 and 40 mm source–detector separations and a placenta depth of 10 mm. Placenta depth has an impact on the detection probability. For a deep placenta, the detectors did not receive a sufficient quantity of photons, which led to a null detection probability. This could be explained by the great absorption of the light by the thick muscle layer. The skin tones have an impact on the detection probability. At a placenta depth of 16 mm, photons are absorbed less by light-toned skin than by dark-toned skin. We also observed that an increase in placental blood volume led to a decrease in detection probability.

In Fig. 7, the detection probabilities of the Mini CYRIL at 780 nm were calculated with an integration time of 10 s and a temporal binning of 10 points. We can observe the effect of the temporal binning, which allows a greater number of photons to be collected by the detectors and leads to an increase in the detection probability. With this hardware configuration, a sufficient quantity of photons is detected for a 30 mm source––detector separation, except for a placenta depth of 20 mm and dark skin tones.

In the Supplementary Material, we represented the detection probabilities of the Mini CYRIL at 780 nm for other configurations (integration times 1 and 5 s), see Figs. S3–S6. We observed that a decrease in the integration time of the device led to a decrease in detection probability. We were not able to calculate detection probabilities at other wavelengths because the optical properties of the solid phantom were only known at 780 nm.

**Fig. 8:**
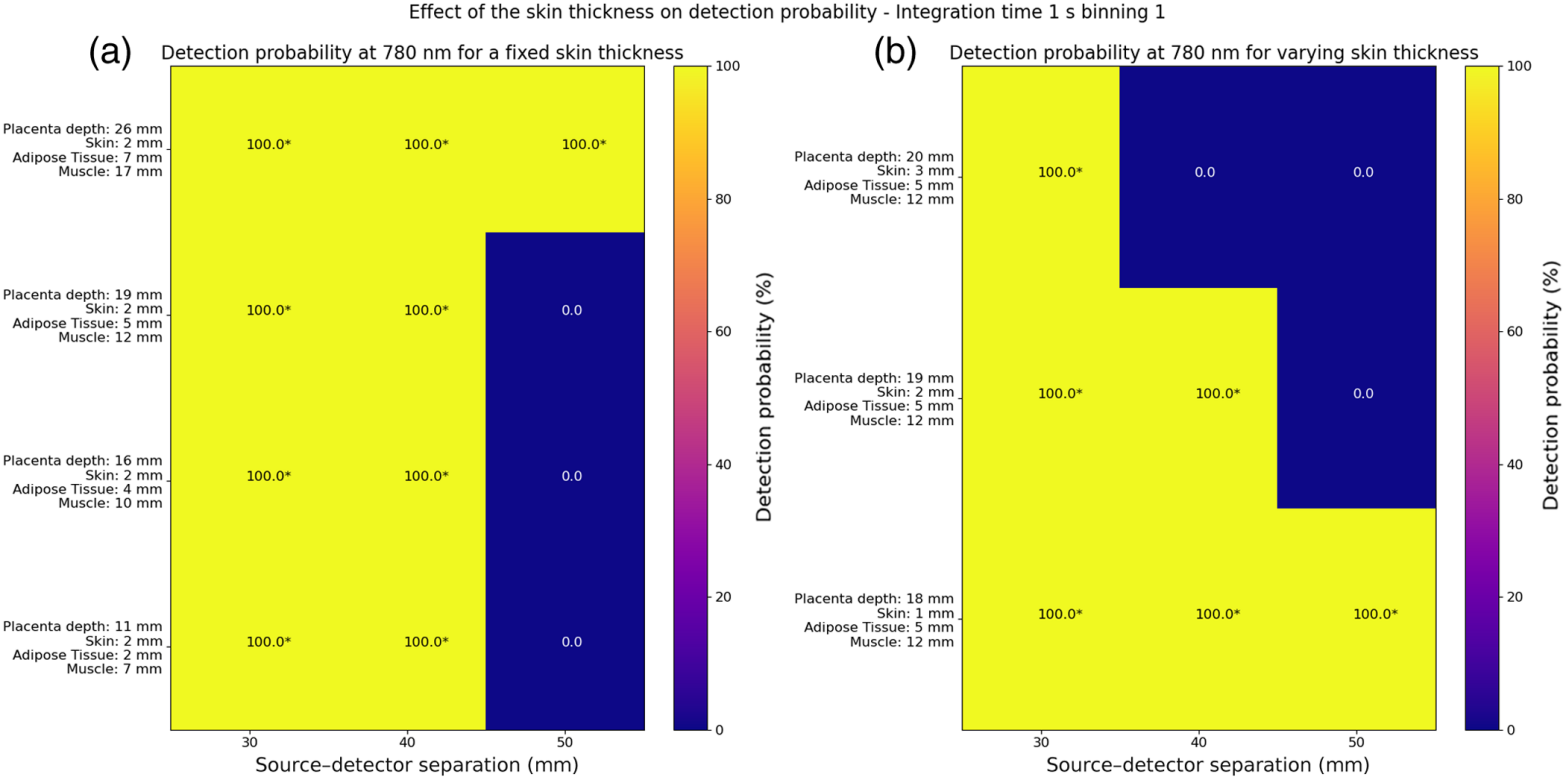
Effect of the skin thickness on the detection probability of the Mini CYRIL at 780 nm for an exposure time of 1 s and no temporal binning. (a) The detection probabilities are for a fixed skin thickness (2 mm). Other tissues have increasing thickness values. (b) The detection probability is represented at 780 nm with varying skin thickness but fixed thickness values for the adipose tissue and the muscle. NIRS signals were simulated for these values, the other parameters were fixed (muscle and placenta blood volume: 35  μMol, muscle ${\mathrm{SatO}}_{2}=60\%$, placenta ${\mathrm{SatO}}_{2}=80\%$ and melanosome volume fraction: 2.55%). Absorption and scattering coefficients of the simulated maternal abdomen are listed below: $\mu_{a}^{\text{skin}}=0.040\;{\mathrm{mm}}^{-1}$, $\mu_{s}^{\text{skin}}=14.38\;{\mathrm{mm}}^{-1}$, $\mu_{a}^{\text{Adipose tissue}}=0.002\;{\mathrm{mm}}^{-1}$, $\mu_{s}^{\text{Adipose tissue}}=13.64\;{\mathrm{mm}}^{-1}$, $\mu_{a}^{\text{Muscle}}=0.0089\;{\mathrm{mm}}^{-1}$, $\mu_{s}^{\text{Muscle}}=8.612\;{\mathrm{mm}}^{-1}$, $\mu_{a}^{\text{Placenta}}=0.0067\;{\mathrm{mm}}^{-1}$, and $\mu_{s}^{\text{Placenta}}=8.814\;{\mathrm{mm}}^{-1}$. Detection probability values were directly derived from the one-sided T-test $p_{\text{value}}$, using the relation $100.(1-p_{\text{value}})$. Values marked with stars * indicated that the null hypothesis has been rejected at the 5% significant level (detection probability >95%), see Eq. (11).

In Fig. 8, we studied the effect of the skin thickness on the detection probabilities of the Mini CYRIL at 780 nm calculated with an integration time of 1 s and no temporal binning. In panel (a), we observed an increase in detection probability for the 50 mm source–detector separation as placenta depth increased, with skin thickness fixed at 2 mm and adipose and muscle thicknesses increasing. In panel (b), we observed a decrease in detection probability for 40 and 50 mm source–detector separations as placenta depth increased, with skin thickness increasing and fixed adipose and muscle thicknesses.

### 3.3 Placenta Sensitivity

**Fig. 9:**
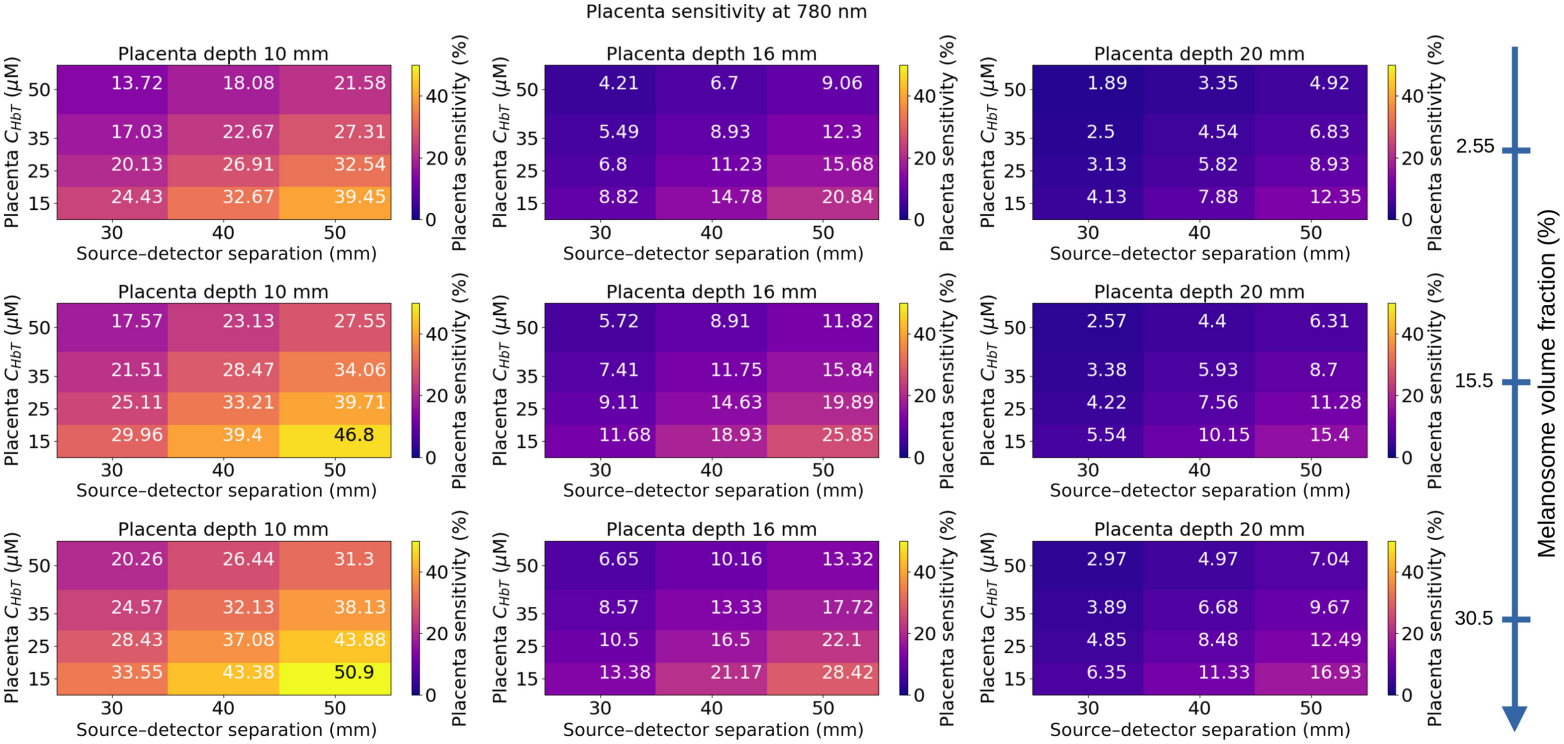
Placenta sensitivity at 780 nm as a function of the placenta blood volume, source–detector separation, melanosome volume fraction, and placenta depth. NIRS signals were simulated for these values, the other parameters were fixed (muscle blood volume: 25  μMol, muscle ${\mathrm{SatO}}_{2}=60\%$, placenta ${\mathrm{SatO}}_{2}=80\%$). Absorption and scattering coefficients of the simulated maternal abdomen are listed below: $\mu_{a}^{\text{skin}}=0.040$, 0.088, and $0.144\;{\mathrm{mm}}^{-1}$ for a melanosome volume fraction of 2.55%, 15.5%, and 30.5%, respectively. $\mu_{s}^{\text{skin}}=14.38\;{\mathrm{mm}}^{-1}$, $\mu_{a}^{\text{Adipose tissue}}=0.002\;{\mathrm{mm}}^{-1}$, $\mu_{s}^{\text{Adipose tissue}}=13.64\;{\mathrm{mm}}^{-1}$, $\mu_{a}^{\text{Muscle}}=0.0069\;{\mathrm{mm}}^{-1}$, $\mu_{s}^{\text{Muscle}}=8.61\;{\mathrm{mm}}^{-1}$, $\mu_{a}^{\text{Placenta}}=0.0049$, 0.0067, 0.0085, and $0.0113\;{\mathrm{mm}}^{-1}$ for $C_{\mathrm{HbT}}^{\text{Placenta}}=15$, 25, 35, and 50  μM, respectively. $\mu_{s}^{\text{Placenta}}=8.81\;{\mathrm{mm}}^{-1}$. For each placenta depth, the thickness of each tissue layer is indicated in Table 4.

**Fig. 10:**
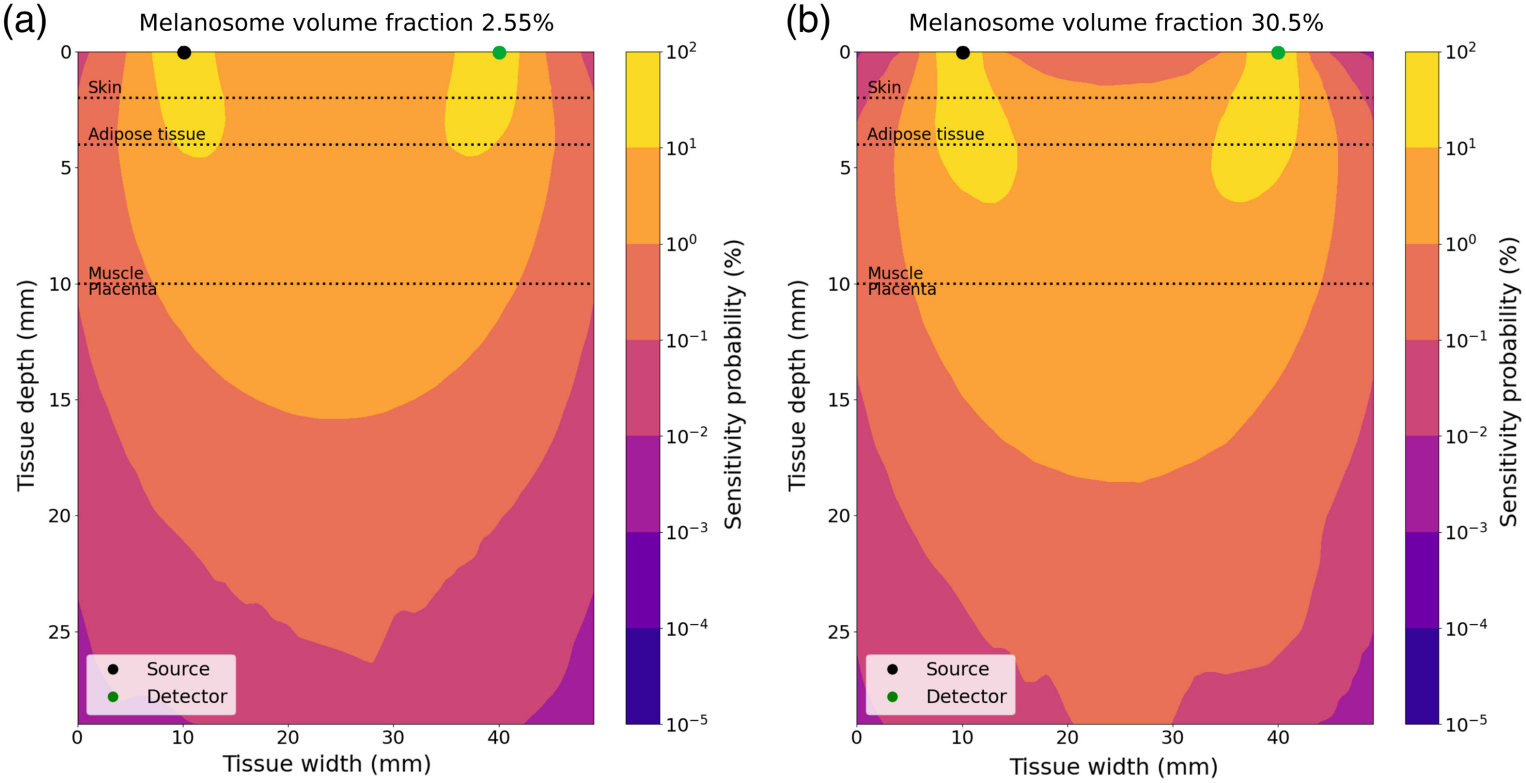
Cross-section of sensitivity probability at 780 nm for a 30 mm source–detector separation. (a) Melanosome volume fraction: 2.55%. (b) Melanosome volume fraction: 30.5%. NIRS signals were simulated for these values; other parameters were the same for graphs (a) and (b) (skin thickness: 2 mm, adipose tissue thickness: 4 mm, muscle thickness: 10 mm, muscle and placenta blood volumes 25  μMol, muscle ${\mathrm{SatO}}_{2}=60\%$, placenta ${\mathrm{SatO}}_{2}=80\%$). Absorption and scattering coefficients of the simulated maternal abdomen are listed below: $\mu_{a}^{\text{skin}}=0.040$ and $0.144\;{\mathrm{mm}}^{-1}$ for a melanosome volume fraction of 2.55 and 30.5%, respectively. $\mu_{s}^{\text{skin}}=14.38\;{\mathrm{mm}}^{-1}$, $\mu_{a}^{\text{Adipose tissue}}=0.002\;{\mathrm{mm}}^{-1}$, $\mu_{s}^{\text{Adipose tissue}}=13.64\;{\mathrm{mm}}^{-1}$, $\mu_{a}^{\text{Muscle}}=0.0069\;{\mathrm{mm}}^{-1}$, $\mu_{s}^{\text{Muscle}}=8.61\;{\mathrm{mm}}^{-1}$, $\mu_{a}^{\text{Placenta}}=0.0067\;{\mathrm{mm}}^{-1}$, and $\mu_{s}^{\text{Placenta}}=8.81\;{\mathrm{mm}}^{-1}$.

In Fig. 9, we represented the placenta sensitivity at 780 nm as a function of the placenta blood volume, source–detector separation, melanosome volume fraction, and placenta depth. The thickness of the layers of the maternal abdomen model used for the calculation of the detection probabilities is shown in Table 4. Placenta sensitivity is lower for a short source–detector separation than for longer ones. For a long source–detector separation, photons have a greater probability to reach the placenta, which translates into an increased placenta sensitivity. Placenta depth has an impact on the placenta sensitivity. For a deep placenta, photons have a lower probability of reaching the placenta, which translates to a reduction of the placenta’s sensitivity. We also observed that placenta sensitivity is lower for very fair skin tones (melanosome volume fraction of 2.55%) than for darker skin tones. This effect can be observed in Fig. 10. Contours of placenta sensitivity probability between 0.1% and 1% are deeper in the tissue for a melanosome volume fraction of 30.5% (dark skin tone) than for a value of 2.55% (very fair skin tone).

Placenta sensitivity values at 840 and 890 nm are also provided in Figs. S7 and S8 in the Supplementary Material. At 840 and 890 nm, placenta sensitivity values are on average 23% and 39% lower than those calculated at 780 nm.

### 3.4 Scanning Probability

**Fig. 11:**
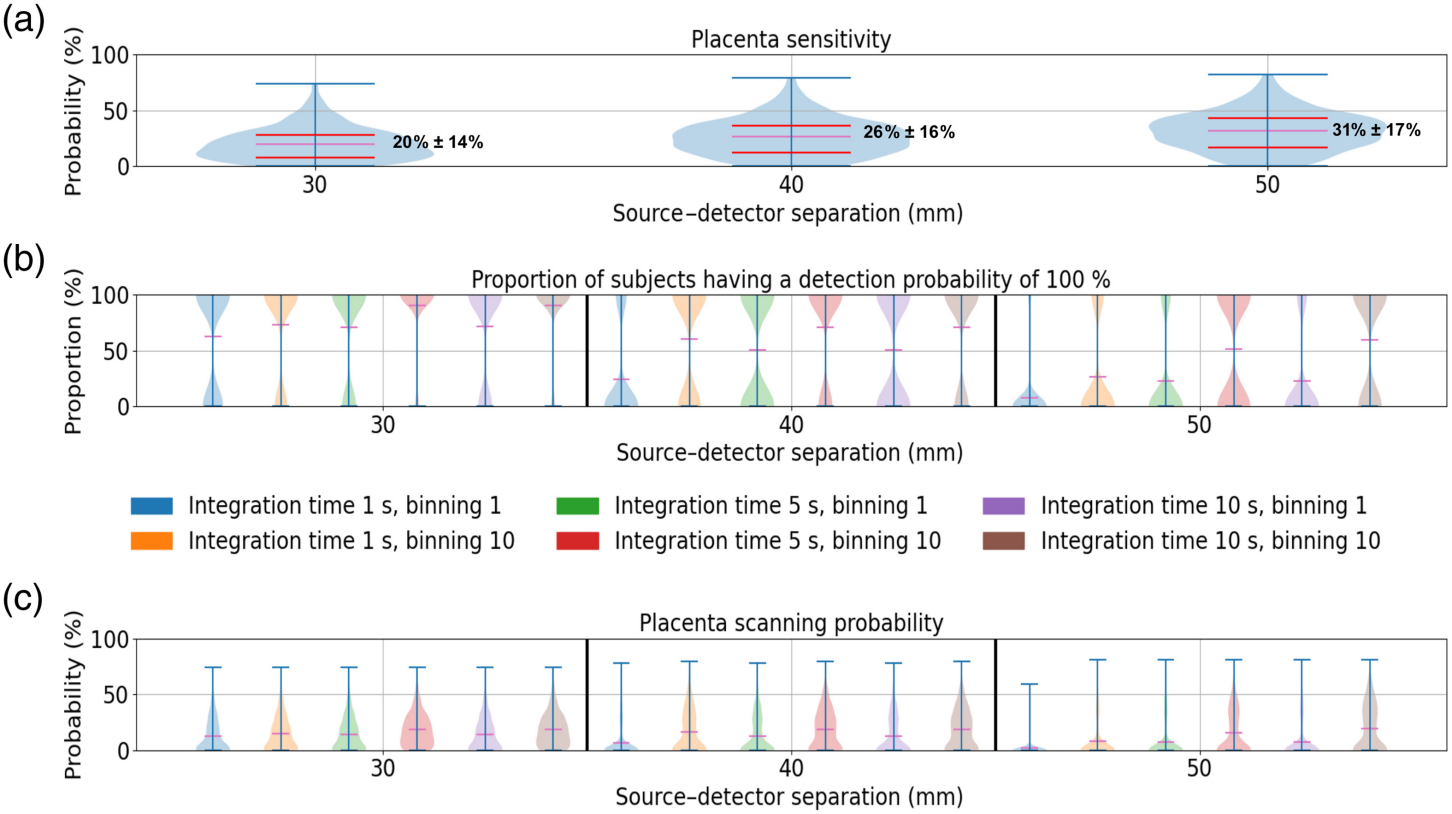
Placenta sensitivity (a), proportion of subjects having a detection probability of 100% (b), and placenta scanning probability (c). The distributions have been simulated for a Mini CYRIL device at 780 nm with 142 healthy subjects. Horizontal magenta lines indicate the mean values of the distribution; red lines indicate the 25th and 75th percentiles. NIRS signals have been simulated based on clinical measurements of tissue thickness and skin tones. Subject tissue thickness has been measured with ultrasound imaging, and the skin tones of the participants were estimated with the Fitzpatrick scale, see Sec. 2.3. For the calculation of the probabilities, total hemoglobin values for muscle and placenta layers were fixed to 35  μM. Oxygen saturation for the muscle and placenta layers was fixed to 60% and 80%, respectively. Absorption and scattering coefficients of the simulated maternal abdomen are listed below: $\mu_{a}^{\text{skin}}=0.040$, 0.088 and $0.144\;{\mathrm{mm}}^{-1}$ for a melanosome volume fraction of 2.55%, 15.5%, and 30.5%, respectively. $\mu_{s}^{\text{skin}}=14.38\;{\mathrm{mm}}^{-1}$, $\mu_{a}^{\text{Adipose tissue}}=0.002\;{\mathrm{mm}}^{-1}$, $\mu_{s}^{\text{Adipose tissue}}=13.64\;{\mathrm{mm}}^{-1}$, $\mu_{a}^{\text{Muscle}}=0.0089\;{\mathrm{mm}}^{-1}$, $\mu_{s}^{\text{Muscle}}=8.61\;{\mathrm{mm}}^{-1}$, $\mu_{a}^{\text{Placenta}}=0.0085\;{\mathrm{mm}}^{-1}$, and $\mu_{s}^{\text{Placenta}}=8.81\;{\mathrm{mm}}^{-1}$.

In Fig. 11, we represented the placenta sensitivity (a), the detection probability (b), and the placenta scanning probability (c) estimated with a Mini CYRIL device at 780 nm on 142 healthy subjects. The probabilities have been calculated with simulated diffuse reflectance values obtained with Redbird software using a realistic modelling of the maternal abdomen. In the model, the thickness of the layers of the maternal abdomen and the Fitzpatrick scale values corresponded to data measured during a clinical examination, see Fig. 3. Total hemoglobin values for muscle and placenta layers were fixed to 35  μM. Oxygen saturation for the muscle and placenta layers was fixed to 60% and 80%, respectively.

In panel (a), placenta sensitivity is lower for detectors close to the light source than for those further away. Note that the large standard deviation values are due to variability in placental depth for 142 subjects, see Fig. 3. In panel (b), the detection probabilities were calculated for several hardware configurations. For each hardware configuration, detection probability values were either 0% or 100%. The width of the violin plots at the lower and upper ends represents the number of subjects with 0% and 100% detection probability, respectively. We can observe the effect of the temporal binning, which allows a greater number of photons to be collected by the detectors and leads to a greater number of subjects having a detection probability of 100%. For a 30 mm source–detector separation and an integration time of 10 s, the detection probability was 100% for almost all subjects with a temporal binning of 10 points. For a 40 mm source–detector separation and an integration time of 10 s, 50% of the subjects had a detection probability of 100% without temporal binning, whereas 70% reached 100% with a temporal binning of 10 points. For a 50 mm source–detector separation, only 30% of the subjects had a detection probability of 100%, indicating that an insufficient number of photons had been detected. In panel (c), the placenta scanning probabilities were obtained by multiplying the placenta sensitivity (a) by the detection probability calculated in graph (b). For a 30 mm source–detector separation, the placenta scanning probabilities were in average close to 20% (mean placenta sensitivity value) because the detection probability of collecting a signal significantly above the noise level was nearly 100% for all subjects. For the 40 and 50 mm source–detector separations, the mean placenta scanning probabilities were lower than the mean placenta sensitivities due to a larger number of subjects with null detection probabilities.

### 3.5 Placental Tissue Oxygenation

**Fig. 12:**
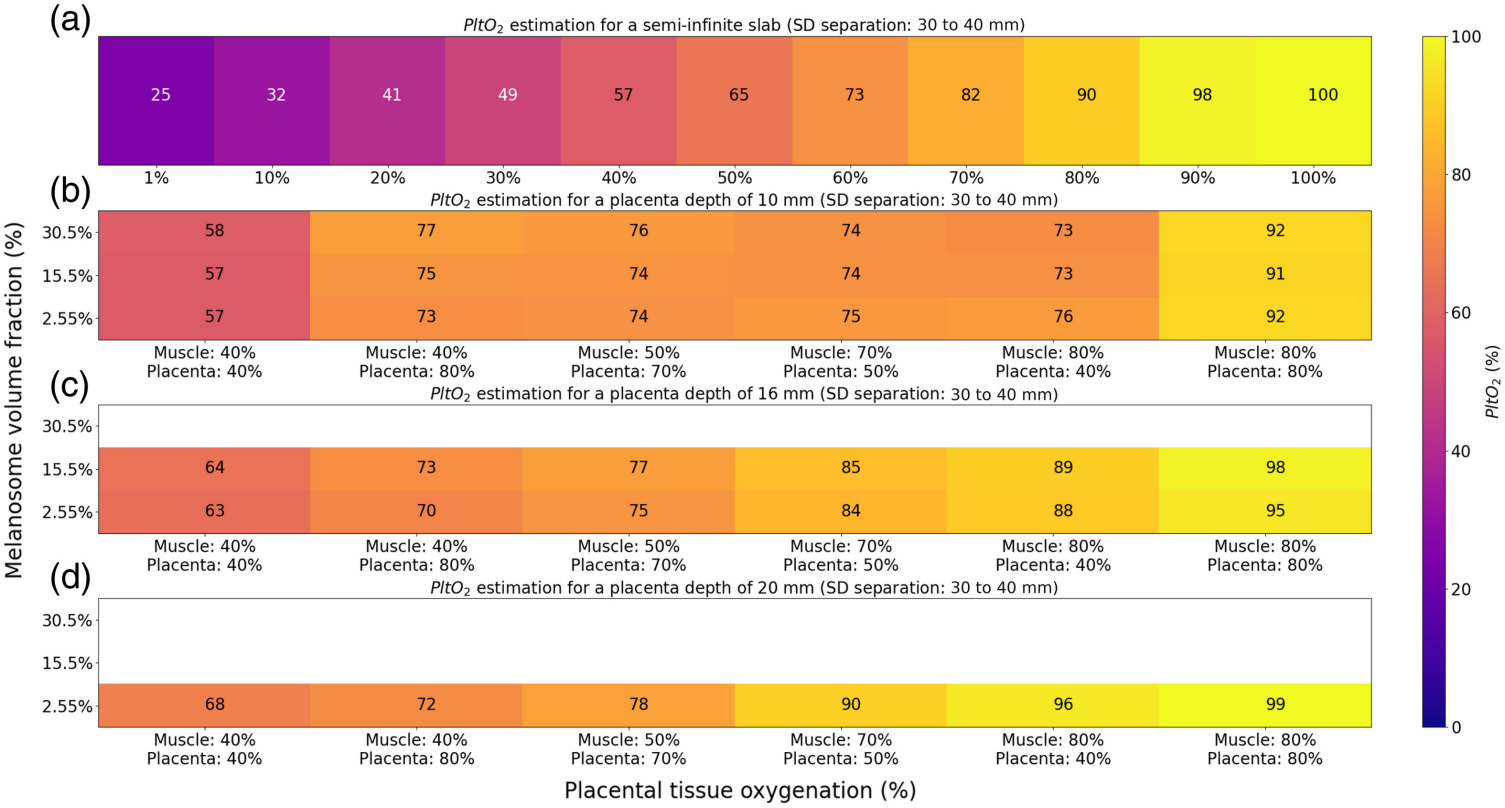
${\mathrm{PltO}}_{2}$ estimation in % using the SRS algorithm with Mini CYRIL using source–detector separations of 30 to 40 mm, an integration time of 10 s and binning of 10 points. (a) ${\mathrm{PltO}}_{2}$ estimation for a homogeneous semi-infinite slab. (b) ${\mathrm{PltO}}_{2}$ estimation for a maternal abdomen model with a placenta depth of 10 mm. (c) ${\mathrm{PltO}}_{2}$ estimation for a maternal abdomen model with a placenta depth of 16 mm. (d) ${\mathrm{PltO}}_{2}$ estimation for a maternal abdomen model with a placenta depth of 20 mm. NIRS signals were simulated for these parameters. The others were fixed (muscle and placenta blood volumes: 35  μM). White rectangles indicate that the ${\mathrm{PltO}}_{2}$ estimation could not be performed because the detectors did not collect a significant amount of photons at 780 nm (the detection probability falls under 95% of the significance level). Absorption and scattering coefficients of the simulated maternal abdomen are listed below: $\mu_{a}^{\text{skin}}=0.040$, 0.088, and $0.144\;{\mathrm{mm}}^{-1}$ for a melanosome volume fraction of 2.55%, 15.5%, and 30.5%, respectively. $\mu_{s}^{\text{skin}}=14.38\;{\mathrm{mm}}^{-1}$, $\mu_{a}^{\text{Adipose tissue}}=0.002\;{\mathrm{mm}}^{-1}$, $\mu_{s}^{\text{Adipose tissue}}=13.64\;{\mathrm{mm}}^{-1}$, $\mu_{a}^{\text{Muscle}}=0.0095$, 0.0092, 0.0086, and $0.0083\;{\mathrm{mm}}^{-1}$ for ${\mathrm{SatO}}_{2}^{\text{Muscle}}=40\%$, 50%, 70%, and 80%, respectively. $\mu_{s}^{\text{Muscle}}=8.61\;{\mathrm{mm}}^{-1}$, $\mu_{a}^{\text{Placenta}}=0.0097$, 0.0094, 0.0088, and $0.0085\;{\mathrm{mm}}^{-1}$ for ${\mathrm{PltO}}_{2}=40\%$, 50%, 70%, and 80%, respectively. $\mu_{s}^{\text{Placenta}}=8.81\;{\mathrm{mm}}^{-1}$.

Placental tissue oxygenation measurements were performed on simulated data with the SRS algorithm using six wavelengths (i.e., 780, 810, 830, 840, 850, and 890 nm) collected with 30 and 40 mm source–detector separations, see Fig. 12. We modelled the Mini-CYRIL device, by considering the detection probability calculated at 780 nm with an integration time of 10 s and a binning of 10 points, see Fig. 7. In panel (a), we represented the estimation of ${\mathrm{PltO}}_{2}$ for a homogeneous semi-infinite slab ($C_{\mathrm{HbT}}=35\;\mu M$ and water volume fraction of 85%). In panels (b)–(d), we showed the estimation of ${\mathrm{PltO}}_{2}$ for models of the maternal abdomen with a placenta depth of 10, 16, and 20 mm, respectively. For panels (b)–(d), three different melanosome volume fractions were considered. We also modeled six different sets of oxygen saturation in the muscle and placenta layers. The total hemoglobin concentrations in the muscle and placenta layers were fixed ($C_{\mathrm{HbT}}=35\;\mu M$). The white rectangles indicate that the ${\mathrm{PltO}}_{2}$ estimation could not be performed because the detectors at 30 or 40 mm separation did not collect a significant amount of photons at 780 nm (the detection probability falls under 95% of the significance level).

The estimation of ${\mathrm{PltO}}_{2}$ in the semi-infinite slab using source–detector separations of 30 to 40 mm is more precise for high ${\mathrm{PltO}}_{2}$ values (on average 9% error for ${\mathrm{PltO}}_{2}\geq 50\%$) than for lower ${\mathrm{PltO}}_{2}$ values (on average 19% error for ${\mathrm{PltO}}_{2}<50\%$). ${\mathrm{PltO}}_{2}$ values can be estimated for all skin tones for placenta depths of 10 mm [see panel (b)]. For deeper placentas, ${\mathrm{PltO}}_{2}$ can only be estimated for light skin tones. Indeed, for dark skin tones, the detection probability dropped to 0% because of the high absorption of light due to melanin. For the placenta depth of 10 and 16 mm [panels (b) and (c)], placenta ${\mathrm{PltO}}_{2}$ values were estimated with low errors when the muscle layer is poorly saturated in $O_{2}$ compared with the placenta. For a placenta depth of 20 mm (graph d), ${\mathrm{PltO}}_{2}$ values presented high errors due to a low placenta sensitivity (sensitivity ≈20%, see Fig. 9).

## 4 Discussion

We developed a digital instrument simulator to evaluate the CW-NIRS device sensitivity for monitoring placental function. With this framework, we proposed an approach to assess placental sensitivity using an adjustable distance CW-NIRS device and solid phantom calibration. The framework optimizes NIRS acquisition parameters (e.g., integration time, source–detector separation) for placental monitoring. This digital instrument simulator can be useful to evaluate the capability of a CW-NIRS device to scan deep layers. Although the methodology has been developed for placenta studies, the use of the framework can be extended to other studies, such as brain functional imaging^20^ and muscle oxygenation.^14^^,^^15^

The CW-NIRS device can be used to monitor the placenta, but precautions must be taken to avoid ambiguities in the analysed signal. Ultrasound imaging is necessary before NIRS acquisition to evaluate the placental scanning probability, which takes into account the detection probability and the placenta sensitivity. The scanning probability can be used to guide the optimization of CW-NIRS acquisition parameters prior to data collection: source–detector separation, integration time (photon collection time), and binning (temporal averaging of the photon collection time). As we can see in Fig. 9, the placenta sensitivity was at best 16% for a placenta depth of 20 mm. CW-NIRS devices are more effective for placentas closer to the surface, with higher sensitivity at specific source–detector separations. For a placenta depth of 10 mm, the placenta sensitivity was at best 43% and 50% for detectors located at 40 and 50 mm from the light source, respectively.

We propose a framework that takes into account both the skin tone and the thickness of the participants. We showed that skin tone has a major impact on detecting a sufficient quantity of signal, see Figs. 6 and 7. Recent studies suggested that a high level of melanin volume fraction in the epidermis leads to measurement biases in pulse oximetry,^51^ spatial frequency domain imaging,^52^ and photo-acoustic imaging.^53^ Feiner et al.^51^ showed that a dark skin tone induces a decrease in the accuracy of pulse oximeters for measuring oxygen saturation. This digital instrument simulator may be used to identify measurement biases in CW-NIRS devices and to propose a correction method for pulse oximetry applications. We also saw that dark tones have a great impact on placenta sensitivity, see Figs. 9 and 10. Counter-intuitively, the placenta sensitivity was greater for darker skins than for lighter skins, whereas the detection probability was greater for lighter skins than for darker skins. Placenta sensitivity refers to the probability that emitted light passes through the placenta for a given source–detector pair. However, it does not account for whether the signal is detected at detector D with a level significantly above the noise; this is captured by the detection probability. In other words, emitted light may reach the placenta, increasing placenta sensitivity, but in some cases, the signal cannot be detected due to high tissue absorption, leading to a decrease in detection probability. This effect is directly linked to the method used to calculate the sensitivity probabilities. Indeed, we used the adjoint method, which involves the voxel-wise multiplication of fluence matrices from both the source and detector positions. For dark skin, the voxel-wise multiplication in the deep layers leads to the calculation of higher values than for lighter skin. However, as the detection probability was lower for subjects with dark skin (see Fig. 7), the values calculated for deep layers do not correspond to an interpretable physical signal. This result indicates that the sensitivity probabilities should not be used without the consideration of the detection probability. To address the limitations in calculating placenta sensitivity, another approach should be considered for computing sensitivity probability volumes, such as the Monte Carlo–based photon replay method.^43^ This approach records the pseudo-random number generator seeds for each detected photon in the Monte Carlo simulation. These photons are then “replayed” to directly generate the sensitivity probability volumes. However, the method is more time-consuming than the approach used in this study, and it requires a large number of emitted photons to reduce stochastic noise because the replay method relies only on detected photons.

We showed that skin thickness has a major impact on detecting a sufficient signal, indicating that robust skin modeling is essential for the analysis of NIRS signals, see Fig. 8. We observed an increase in detection probability as placenta depth increased, with fixed skin thickness and increasing adipose and muscle thicknesses. To explain these results, skin, adipose, muscle, and placenta sensitivities can be examined (see panels (a), (c), (e), and (g) in Fig. S9 in the Supplementary Material). With fixed skin thickness, skin sensitivity remained constant. However, adipose sensitivity increased (up to 60%), whereas muscle and placenta sensitivities showed a slight decrease. These results indicate that light preferentially passes through the adipose tissue rather than the muscle and placenta layers. As adipose tissue is less absorbing than muscle and placenta, more photons have the potential to reach the detectors. We also observed a decrease in detection probability as placenta depth increased, with skin thickness increasing and fixed adipose and muscle thicknesses. To explain these results, skin, adipose, muscle, and placenta sensitivities can be examined (see panels (b), (d), (f), and (h) in Fig. S9 in the Supplementary Material). With increasing skin thickness and fixed adipose and muscle thicknesses, skin sensitivity increased, adipose sensitivity decreased, and muscle and placenta sensitivities increased. Once the light penetrated the skin and adipose tissue layers, it was absorbed in the muscle and placenta layers (increased sensitivity). Similar effects were also observed with increasing melanosome volume fraction while keeping tissue thickness constant (see Figs. 6 and 7). These results indicate that skin thickness and skin tone have a filtering effect on the NIRS signal, blocking backscattered light (decreasing detection probability) and leading to greater absorption in deeper tissues (increasing placenta sensitivity).

To enable the clinical use of this digital instrument simulator, precise quantification of skin tone and thickness is required. Skin tone is assessed using the Fitzpatrick scale, a subjective method susceptible to inter-observer variability. In future studies, quantitative measurements of melanin volume fraction could be estimated with diffuse reflectance spectroscopy.^54^ Skin thickness measurements are obtained via ultrasound imaging. However, the precision of these measurements is constrained by the limited resolution of standard clinical ultrasound devices (0.3 to 0.5 mm^55^), which limits their effectiveness in measuring thin anatomical structures such as the skin. Although the skin thickness measurements are not exact, they are of the same order of magnitude as those reported by Oltulu, who accurately measured skin thickness by histopathological analysis.^41^

In Figs. 6–8, detection probability values were either 0% or 100%. These binary results indicated that the mean diffuse reflectance values were significantly below or above the noise level. This is linked to the large sample size (n=10,000) used for the calculation of the $p_{\text{values}}$. Indeed, with a large sample size, the standard error ($\frac{\sigma }{\sqrt{n}}$, with σ the standard deviation of the sample, and n=10,000 the sample size) becomes small. Thus, the statistical test is more likely to produce $p_{\text{values}}$ of 0 (below machine precision) or 1,^56^ leading to detection probabilities of 100% or 0%, respectively ($100.(1-p_{\text{value}})$). The effect of sample size on detection probability is shown in Fig. S12 in the Supplementary Material.

In this digital instrument simulator, all simulations have been done at 780 nm. With other wavelengths, placenta sensitivities are notably different, see Figs. S7 and S8 in the Supplementary Material. We choose this wavelength because it is usually included in NIRS devices and the optical properties of the solid phantom used in the calibration procedure are known for this wavelength. In future work, we plan to evaluate the detection probability of the Mini-CYRIL device across all wavelengths. This requires a broadband calibration with a multilayer phantom of known optical properties at all device wavelengths. For this purpose, a multilayer liquid phantom based on blood, water, and intralipid can be used.^57^

In this study, we present a method for assessing placental sensitivity using the Mini CYRIL device, which integrates a broadband light source and a spectrometer at different source–detector separations.^26^ This was used as an example instrument to test our simulation methods. We believe that, and as shown by our simulations, placental scanning probability could be enhanced using an optimized device, such as an LED-based multiwavelength NIRS system, capable of delivering coherent, higher optical power, and integrating detectors with higher quantum efficiency and sensitivity.

We showed in Fig. 12 that placental tissue oxygenation can be precisely measured with the SRS algorithm using 30 to 40 mm source–detector separations under specific conditions. Precise measurements can be made for placentas close to the surface, when the muscle tissue oxygenations are lower than those of the placenta. The estimation of placental tissue oxygenation showed large errors when the muscle and placenta layers had low oxygen saturation. We also observed this effect when modeling a semi-infinite slab, see Fig. 12(a).

In the future, we plan to extend this work and propose a method for measuring the oxygen saturation in the placenta layer. We plan to work on a new spatially resolved spectroscopy method to overcome the limitations of the current algorithm.^22^ Indeed, this method is adapted for homogeneous media but introduces large errors in layered media, see Fig. 12. For this, we can take profit of the estimation of the placenta sensitivity as a prior information of the mixing contribution of the collected signal. We can also take profit of the algorithm proposed by Wang et al.,^16^ who estimated the oxygen saturation in the placenta by fitting measured with theoretical spectra obtained with a multilayered model for light propagation.^58^^,^^59^ The estimation of the placental tissue oxygenation could also be improved with the consideration of water and fat proportion, as proposed by Kovacsova et al.^60^ In this work, the authors proposed estimating tissue oxygen saturation using a broadband multidistance oximetry algorithm based on fitting the gradient of light attenuation versus distance measured with broadband NIRS. As our pipeline is based on the Redbird toolbox,^29^^,^^30^ we can also use Redbird’s inverse solver to recover distributions of unknown optical properties by fitting forward simulations to measured data.

Placental sensitivity and detection probabilities can also be calculated with Monte Carlo simulations, for example, MCX.^31^ One of the differences between Redbird and MCX lies in the estimation of diffuse reflectance. In MCX, this value is directly related to the number of photons exiting the volume, which requires launching a large number of photons to reduce stochastic noise in the estimated data. With Redbird, the diffuse reflectance is calculated from the fluence values and is free of noise because this toolbox is based on a numerical solution of the diffusion equation using a finite-element method.

We simulated with MCX the 142 maternal abdomens segmented in our study and calculated the corresponding placental sensitivities and detection probabilities, see Fig. S11 in the Supplementary Material. We obtained similar results with Redbird and MCX, the mean absolute differences between the distributions obtained with Redbird and Monte Carlo simulations were 4.18% for placental sensitivities and 6.69% for detection probabilities, see Tables S1 and S2 in the Supplementary Material.

Redbird is a practical toolbox for modelling light propagation in biological tissues, is free of noise, and is about 100 times faster than GPU-based Monte Carlo MMC/MCX software.^31^^,^^32^ However, Redbird is only valid in high-scattering media, where the reduced scattering coefficient $\mu_{s}^{\prime }$ (in ${\mathrm{mm}}^{-1}$) is much greater than the absorption coefficient $\mu_{a}^{\prime }$ (in ${\mathrm{mm}}^{-1}$). Using Redbird in low-scattering media such as water or cerebrospinal fluid may produce biased results.

## 5 Conclusion

We developed a digital instrument simulator to evaluate the sensitivity of CW-NIRS devices for monitoring the placental function. This tool can be used to estimate the mixing contributions of the collected signal and could be used to optimize acquisition parameters such as the integration time and the temporal binning of CW-NIRS devices.

## Supplementary Material

10.1117/1.JBO.31.2.027003.s01

## Data Availability

In support of open science, the code presented in this article is publicly available on https://github.com/CCaredda/CW-NIRS_Placenta_Sensitivity.
