# Supplementary material for: Digital instrument simulator platform to support the development of noninvasive optical NIR device for placenta monitoring
Source: J Biomed Opt. 2026 Feb 20;31(2):027003. doi: 10.1117/1.JBO.31.2.027003 (PMC12923275; doi:10.1117/1.JBO.31.2.027003)
Supplement: Supplementary file 1 [file JBO_031_027003_SD001.pdf]

Digital Instrument Simulator Platform to Support the Development of Non-Invasive Optical NIR Device for placenta monitoring

Charly Caredda[a*], Frédéric Lange[b], Niccole Ranaei-Zamani[c], Uzair Hakim[b], Olayinka Kowobari[c], Dimitrios Siassakos[c], Sara Hillman[c], Anna L David[c], Subhabrata Mitra[c], Ilias Tachtsidis[b*]

[a]INSA-Lyon, Université Claude Bernard Lyon 1, UJM-Saint Etienne, CNRS, Inserm, CREATIS UMR 5220, U1294, F69100, Lyon, France

[b]Department of Medical Physics and Biomedical Engineering, University College London, London, UK

[c]EGA Institute for Women's Health, University College London, London, United Kingdom

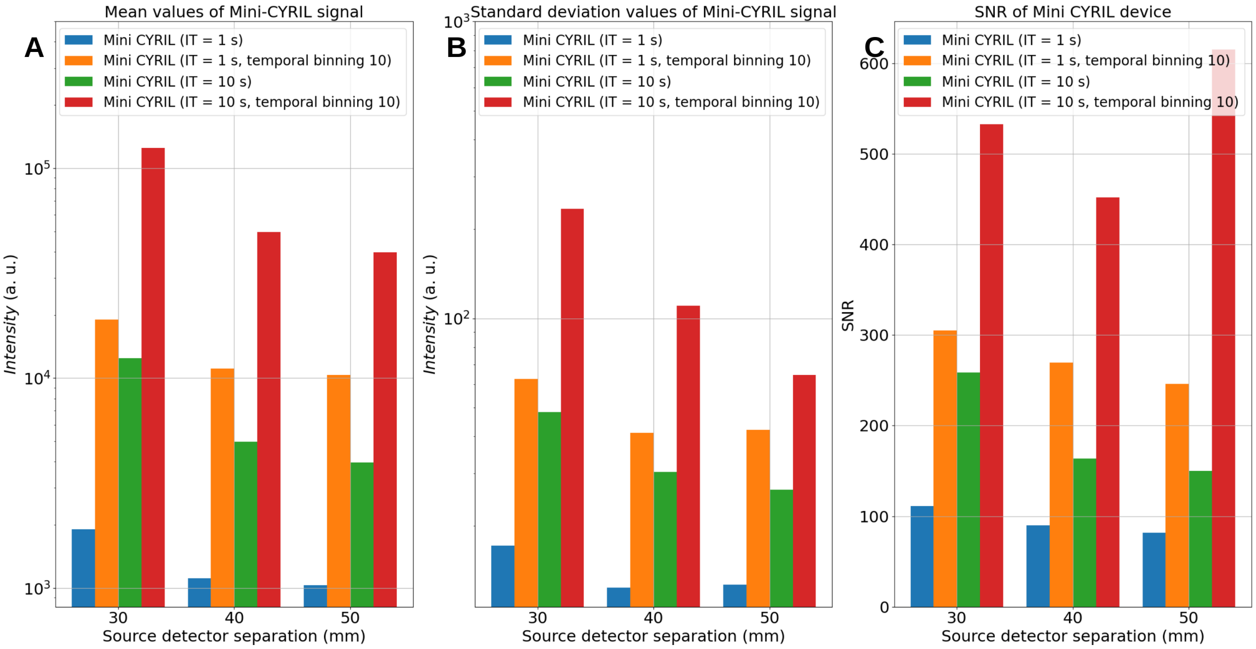

Fig S1 Mean (A), standard deviation (B) and signal to noise ratio-SNR (C) values measured with Mini-CYRIL at 780 nm with the mutli-layered solid phantom.

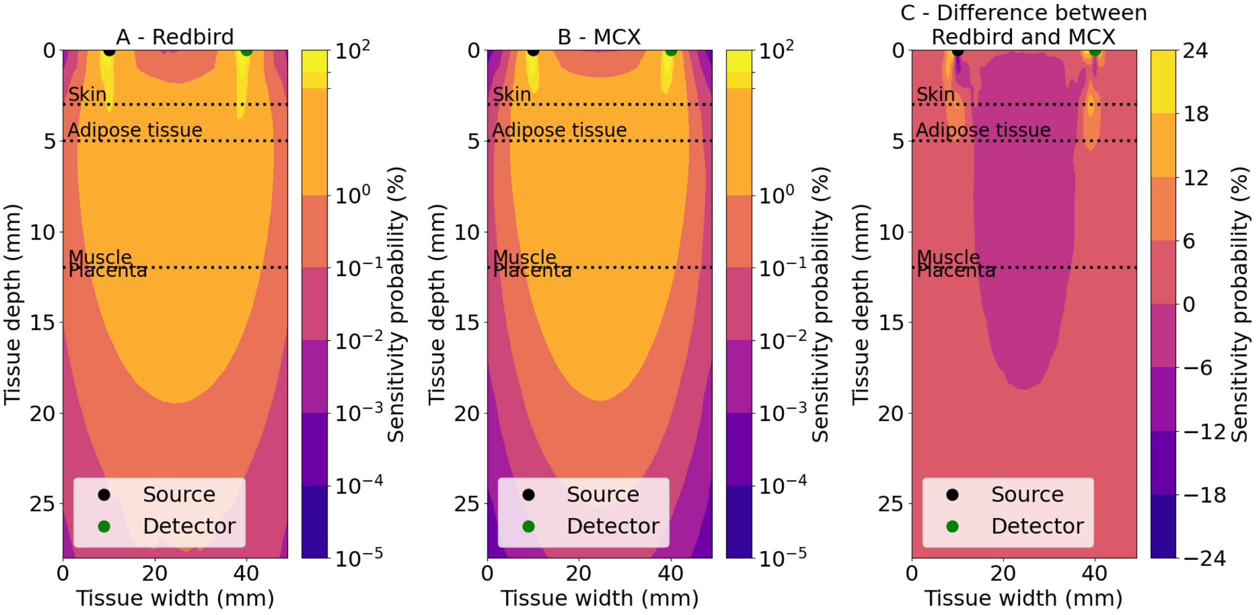

Fig S2 Cross-section of sensitivity probability at 780 nm for a source-detector separation of 30 mm calculated with Redbird (A) and MCX (B). C - Difference between the sensitivity maps calculated with Redbird and MCX. NIRS signals were simulated for a placenta depth of 20 mm (skin thickness: 3 mm, adipose tissue thickness: 5 mm, muscle thickness: 12 mm), a melanosome volume fraction of 15.5%, muscle and placenta blood volume of 25 μM and 35 μM, respectively, a muscle and placenta oxygen saturation of 60 and 80%.

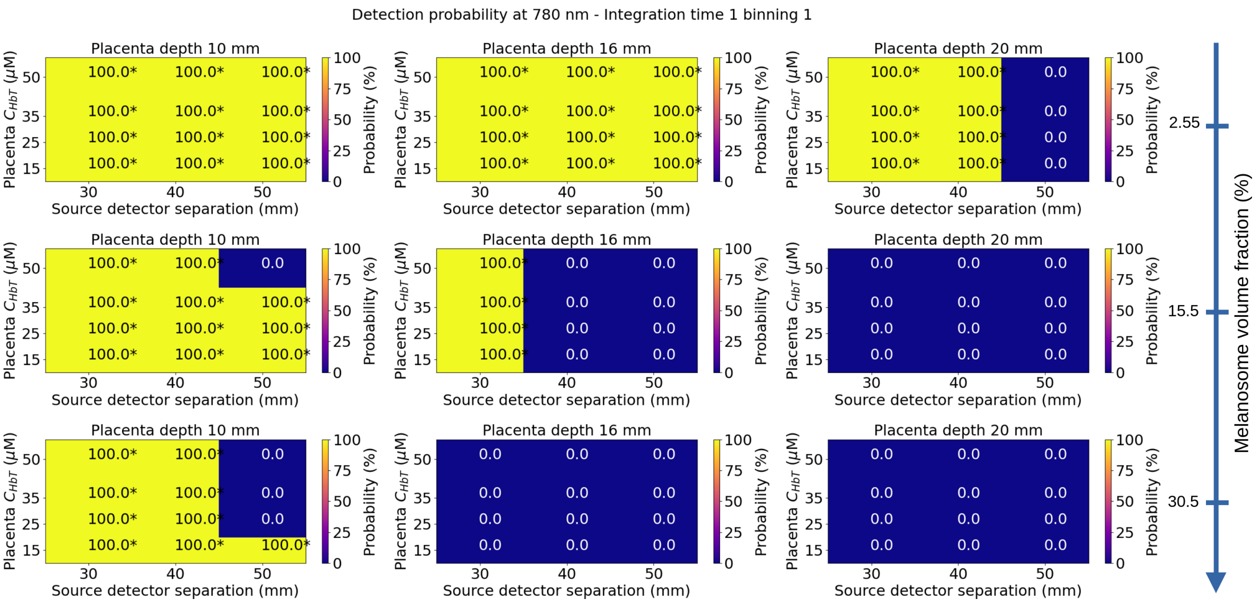

Fig S3 Detection probability of the Mini CYRIL at 780 nm for an exposure time of 1 s and no temporal binning as function of the placenta blood volume, source-detector separation, melanosome volume fraction and placenta depth. The other parameters were fixed (muscle blood volume: 25 μMol, muscle $SatO_2 = 60\%$, placenta $SatO_2 = 80\%$). Absorption and scattering coefficients of the simulated maternal abdomen are listed below: $\mu_a^{\text{skin}} = 0.040$, 0.088 and 0.144 mm^{-1} for a melanosome volume fraction of 2.55, 15.5 and 30.5%, respectively. $\mu_s^{\text{skin}} = 14.38$ mm^{-1}, $\mu_a^{\text{Adipose tissue}} = 0.002$ mm^{-1}, $\mu_s^{\text{Adipose tissue}} = 13.64$ mm^{-1}, $\mu_a^{\text{Muscle}} = 0.0069$ mm^{-1}, $\mu_s^{\text{Muscle}} = 8.61$ mm^{-1}, $\mu_a^{\text{Placenta}} =$ 0.0049, 0.0067, 0.0085 and 0.0113 mm^{-1} for $C_{HbT}^{\text{Placenta}} = 15$, 25, 35 and 50 μM, respectively. $\mu_s^{\text{Placenta}} = 8.81$ mm^{-1}.

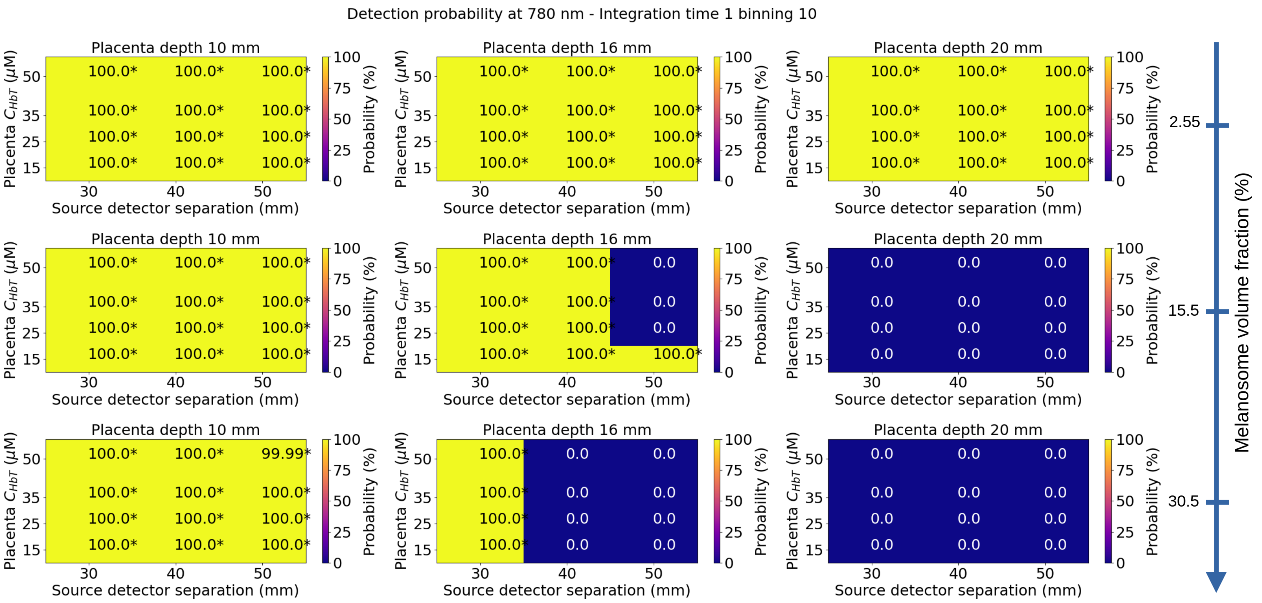

Fig S4 Detection probability of the Mini CYRIL at 780 nm for an exposure time of 1 s and a temporal binning of 10 s as function of the placenta blood volume, source-detector separation, melanosome volume fraction and placenta depth. The other parameters were fixed (muscle blood volume: $25\ \mu Mol$, muscle $SatO_2 = 60\%$, placenta $SatO_2 = 80\%$). Absorption and scattering coefficients of the simulated maternal abdomen are listed below: $\mu_a^{\text{skin}} = 0.040$, 0.088 and 0.144 mm$^{-}1$ for a melanosome volume fraction of 2.55, 15.5 and 30.5%, respectively. $\mu_s^{\text{skin}} = 14.38$ mm^{-1}, $\mu_a^{\text{Adipose tissue}} = 0.002$ mm^{-1}, $\mu_s^{\text{Adipose tissue}} = 13.64$ mm^{-1}, $\mu_a^{\text{Muscle}} = 0.0069$ mm^{-1}, $\mu_s^{\text{Muscle}} = 8.61$ mm^{-1}, $\mu_a^{\text{Placenta}} = 0.0049$, 0.0067, 0.0085 and 0.0113 mm^{-1} for $C_{HbT}^{\text{Placenta}} = 15$, 25, 35 and $50\ \mu M$, respectively. $\mu_s^{\text{Placenta}} = 8.81$ mm^{-1}.

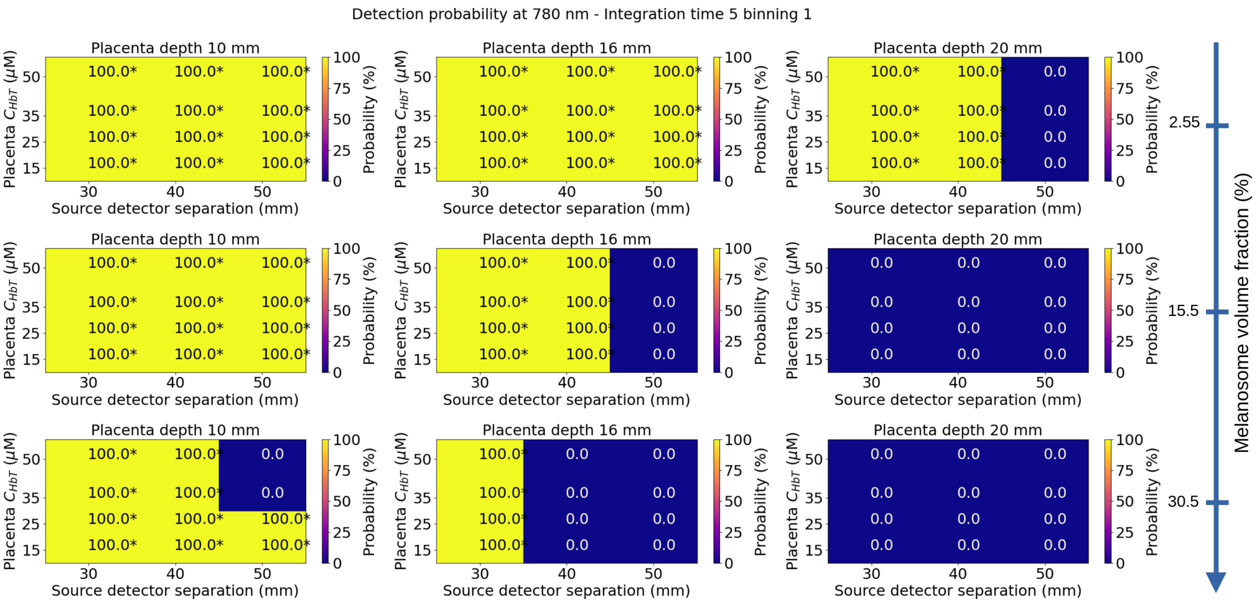

Fig S5 Detection probability of the Mini CYRIL at 780 nm for an exposure time of 5 s and no temporal binning as function of the placenta blood volume, source-detector separation, melanosome volume fraction and placenta depth. The other parameters were fixed (muscle blood volume: 25 μMol, muscle $SatO_2 = 60\%$, placenta $SatO_2 = 80\%$). Absorption and scattering coefficients of the simulated maternal abdomen are listed below: $\mu_a^{\text{skin}} = 0.040$, 0.088 and 0.144 mm-1 for a melanosome volume fraction of 2.55, 15.5 and 30.5%, respectively. $\mu_s^{\text{skin}} = 14.38$ mm^{-1}, $\mu_a^{\text{Adipose tissue}} = 0.002$ mm^{-1}, $\mu_s^{\text{Adipose tissue}} = 13.64$ mm^{-1}, $\mu_a^{\text{Muscle}} = 0.0069$ mm^{-1}, $\mu_s^{\text{Muscle}} = 8.61$ mm^{-1}, $\mu_a^{\text{Placenta}} = 0.0049, 0.0067, 0.0085$ and 0.0113 mm^{-1} for $C_{HbT}^{\text{Placenta}} = 15$, 25, 35 and 50 μM, respectively. $\mu_s^{\text{Placenta}} = 8.81$ mm^{-1}.

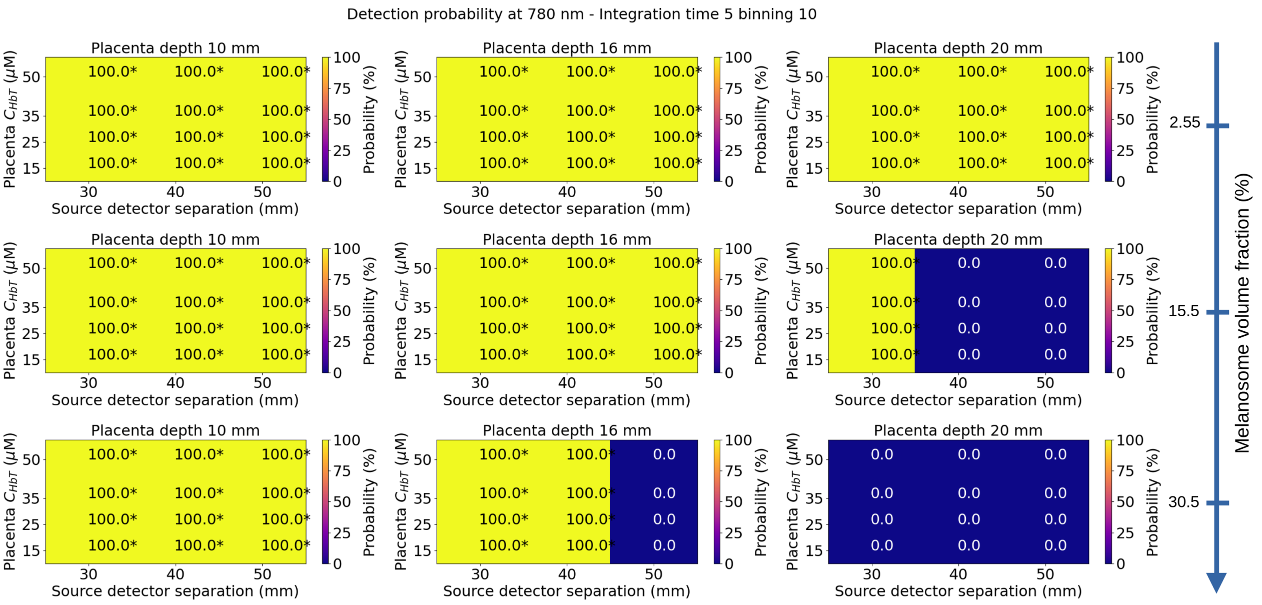

Fig S6 Detection probability of the Mini CYRIL at 780 nm for an exposure time of 5 s and a temporal binning of 10 s as function of the placenta blood volume, source-detector separation, melanosome volume fraction and placenta depth. The other parameters were fixed (muscle blood volume: 25 μMol, muscle $SatO_2 = 60\%$, placenta $SatO_2 = 80\%$). Absorption and scattering coefficients of the simulated maternal abdomen are listed below: $\mu_a^{\text{skin}} = 0.040$, 0.088 and 0.144 mm$^-1$ for a melanosome volume fraction of 2.55, 15.5 and 30.5%, respectively. $\mu_s^{\text{skin}} = 14.38$ mm^{-1}, $\mu_a^{\text{Adipose tissue}} = 0.002$ mm^{-1}, $\mu_s^{\text{Adipose tissue}} = 13.64$ mm^{-1}, $\mu_a^{\text{Muscle}} = 0.0069$ mm^{-1}, $\mu_s^{\text{Muscle}} = 8.61$ mm^{-1}, $\mu_a^{\text{Placenta}} = 0.0049$, 0.0067, 0.0085 and 0.0113 mm^{-1} for $C_{HbT}^{\text{Placenta}} = 15$, 25, 35 and 50 μM, respectively. $\mu_s^{\text{Placenta}} = 8.81$ mm^{-1}.

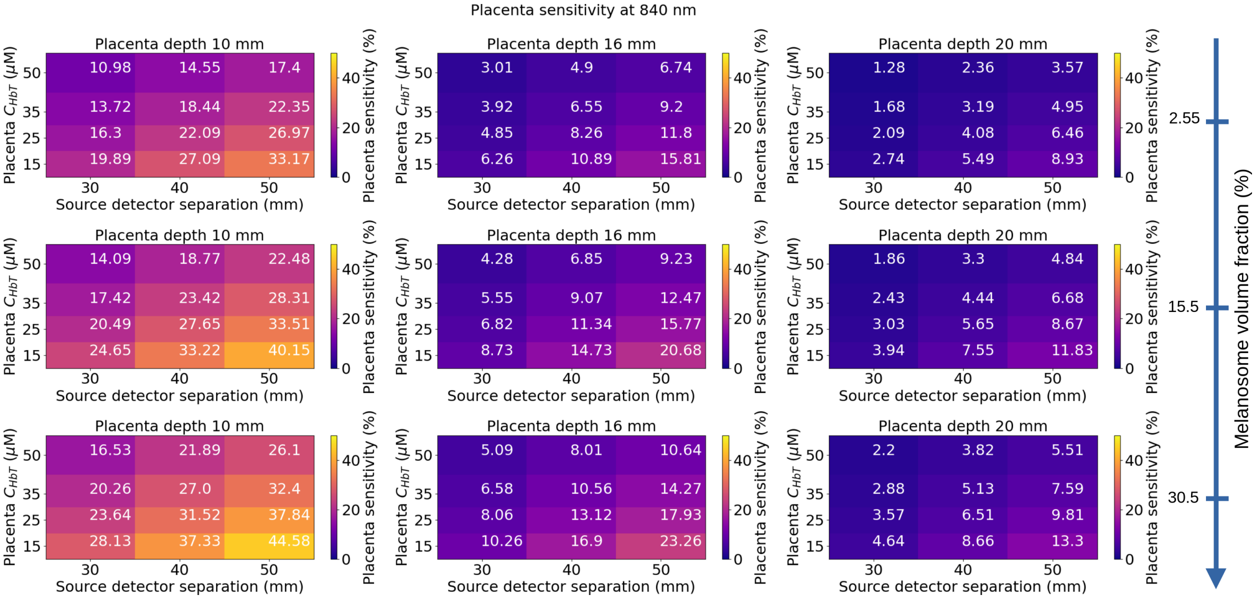

Fig S7 Placenta sensitivity at 840 nm as function of the placenta blood volume, source-detector separation, melanosome volume fraction and placenta depth. The other parameters were fixed (muscle blood volume: 25 μMol, muscle $SatO_2$ = 60%, placenta $SatO_2$ = 80%). Absorption and scattering coefficients of the simulated maternal abdomen are listed below: μ_a^{skin} = 0.0321, 0.0706 and 0.1151 mm^{-}1 for a melanosome volume fraction of 2.55, 15.5 and 30.5%, respectively. $\mu_s^{\text{skin}} = 12.94$ mm^{-1}, $\mu_a^{\text{Adipose tissue}} = 0.0034$ mm^{-1}, $\mu_s^{\text{Adipose tissue}} = 12.98$ mm^{-1}, $\mu_a^{\text{Muscle}} = 0.0082$ mm^{-1}, $\mu_s^{\text{Muscle}} = 8.041$ mm^{-1}, $\mu_a^{\text{Placenta}} = 0.0068, 0.0090, 0.0112, 0.0145$ mm^{-1} for $C_{HbT}^{\text{Placenta}} = 15$, 25, 35 and 50 μM, respectively. $\mu_s^{\text{Placenta}} = 7.930$ mm^{-1}.

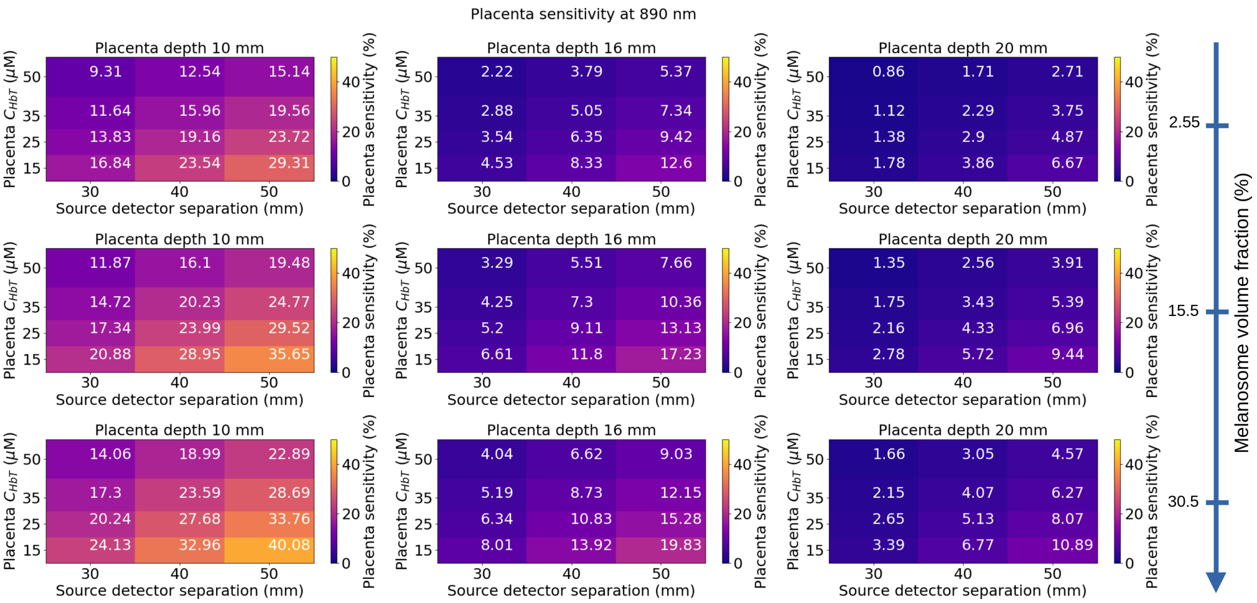

Fig S8 Placenta sensitivity at 890 nm as function of the placenta blood volume, source-detector separation, melanosome volume fraction and placenta depth. The other parameters were fixed (muscle blood volume: 25 μMol, muscle $SatO_2 = 60\%$, placenta $SatO_2 = 80\%$). Absorption and scattering coefficients of the simulated maternal abdomen are listed below: $\mu_a^{\text{skin}} = 0.0269$, 0.0593 and 0.0967 mm$^-1$ for a melanosome volume fraction of 2.55, 15.5 and 30.5%, respectively. $\mu_s^{\text{skin}} = 11.92$ mm^{-1}, $\mu_a^{\text{Adipose tissue}} = 0.0054$ mm^{-1}, $\mu_s^{\text{Adipose tissue}} = 12.989$ mm^{-1}, $\mu_a^{\text{Muscle}} = 0.0103$ mm^{-1}, $\mu_s^{\text{Muscle}} = 7.62$ mm^{-1}, $\mu_a^{\text{Placenta}} = 0.0088, 0.0113, 0.0138, 0.0176$ mm^{-1} for $C_{HbT}^{\text{Placenta}} = 15$, 25, 35 and 50 μM, respectively. $\mu_s^{\text{Placenta}} = 7.303$ mm^{-1}.

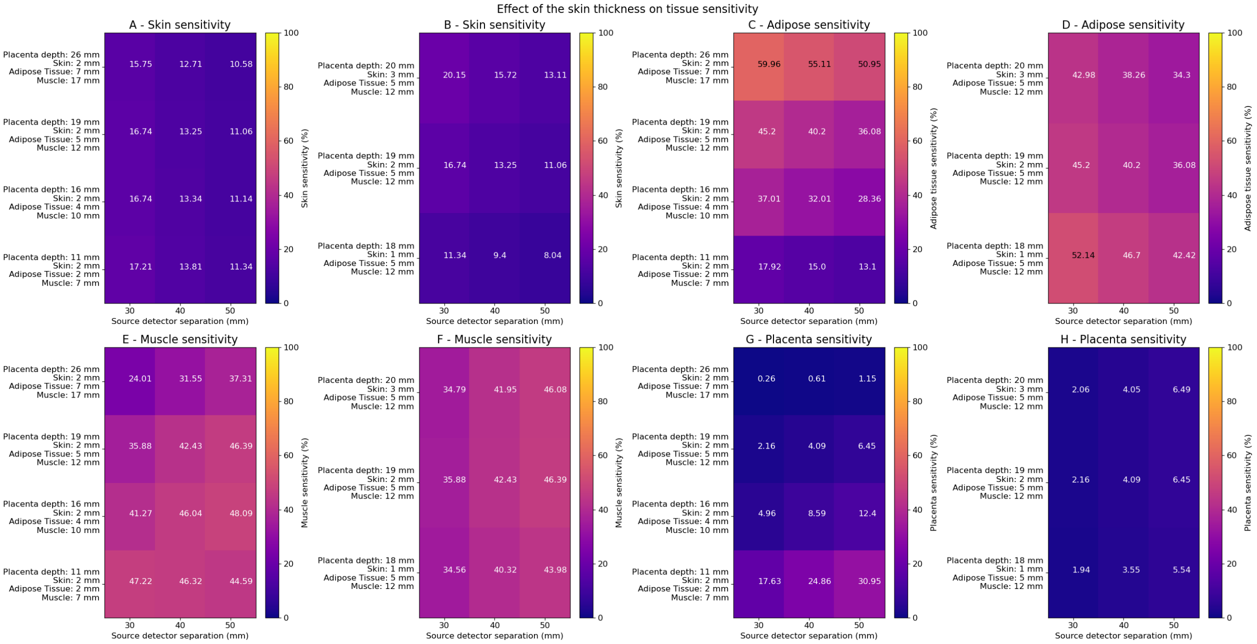

Fig S9 Effect of the skin on tissue sensitivities. A - Skin sensitivity with fixed skin thickness and increasing adipose and muscle thickness. B - Skin sensitivity with increasing skin thickness and fixed adipose and muscle thickness. C - Adipose tissue sensitivity with fixed skin thickness and increasing adipose and muscle thickness. D - Adipose tissue sensitivity with increasing skin thickness and fixed adipose and muscle thickness. E - Muscle sensitivity with fixed skin thickness and increasing adipose and muscle thickness. F - Muscle sensitivity with increasing skin thickness and fixed adipose and muscle thickness. G - Placenta sensitivity with fixed skin thickness and increasing adipose and muscle thickness. H - Placenta sensitivity with increasing skin thickness and fixed adipose and muscle thickness. The other parameters were fixed (muscle blood volume: 25 μMol, muscle $SatO_2 = 60\%$, placenta $SatO_2 = 80\%$ and melanosome volume fraction: 2.55%). Absorption and scattering coefficients of the simulated maternal abdomen are listed below: $\mu_a^{\text{skin}} = 0.040$ mm^{-1}, $\mu_s^{\text{skin}} = 14.38$ mm^{-1}, $\mu_a^{\text{Adipose tissue}} = 0.002$ mm^{-1}, $\mu_s^{\text{Adipose tissue}} = 13.64$ mm^{-1}, $\mu_a^{\text{Muscle}} = 0.0089$ mm^{-1}, $\mu_s^{\text{Muscle}} = 8.61$ mm^{-1}, $\mu_a^{\text{Placenta}} = 0.0067$ mm^{-1}, $\mu_s^{\text{Placenta}} = 8.81$ mm^{-1}.

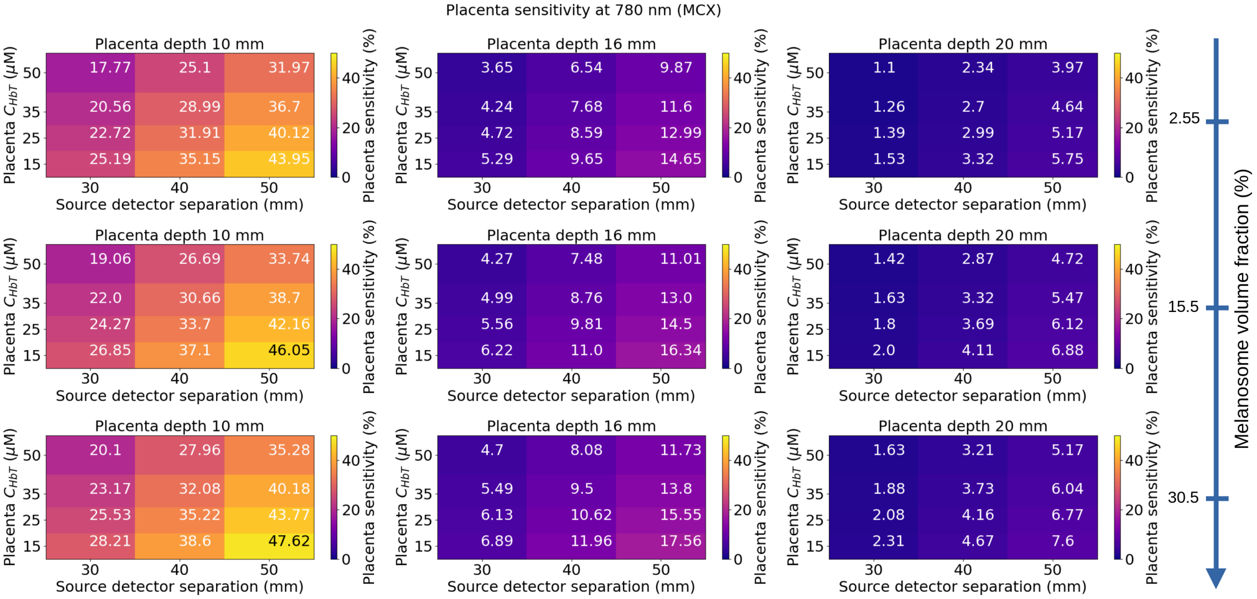

Fig S10 Placenta sensitivity at 780 nm as function of the placenta blood volume, source-detector separation, melanosome volume fraction and placenta depth. NIRS signals were simulated for these values (Data have been calculated with Monte Carlo simulations MCX). The other parameters were fixed (muscle blood volume: 25 μMol, muscle $SatO_2 = 60\%$, placenta $SatO_2 = 80\%$). Absorption and scattering coefficients of the simulated maternal abdomen are listed below: $\mu_a^{\text{skin}} = 0.040$, 0.088 and 0.144 mm$^-1$ for a melanosome volume fraction of 2.55, 15.5 and 30.5%, respectively. $\mu_s^{\text{skin}} = 14.38$ mm^{-1}, $\mu_a^{\text{Adipose tissue}} = 0.002$ mm^{-1}, $\mu_s^{\text{Adipose tissue}} = 13.64$ mm^{-1}, $\mu_a^{\text{Muscle}} = 0.0069$ mm^{-1}, $\mu_s^{\text{Muscle}} = 8.61$ mm^{-1}, $\mu_a^{\text{Placenta}} = 0.0049, 0.0067, 0.0085$ and 0.0113 mm^{-1} for $C_{HbT}^{\text{Placenta}} = 15$, 25, 35 and 50 μM, respectively. $\mu_s^{\text{Placenta}} = 8.81$ mm^{-1}.

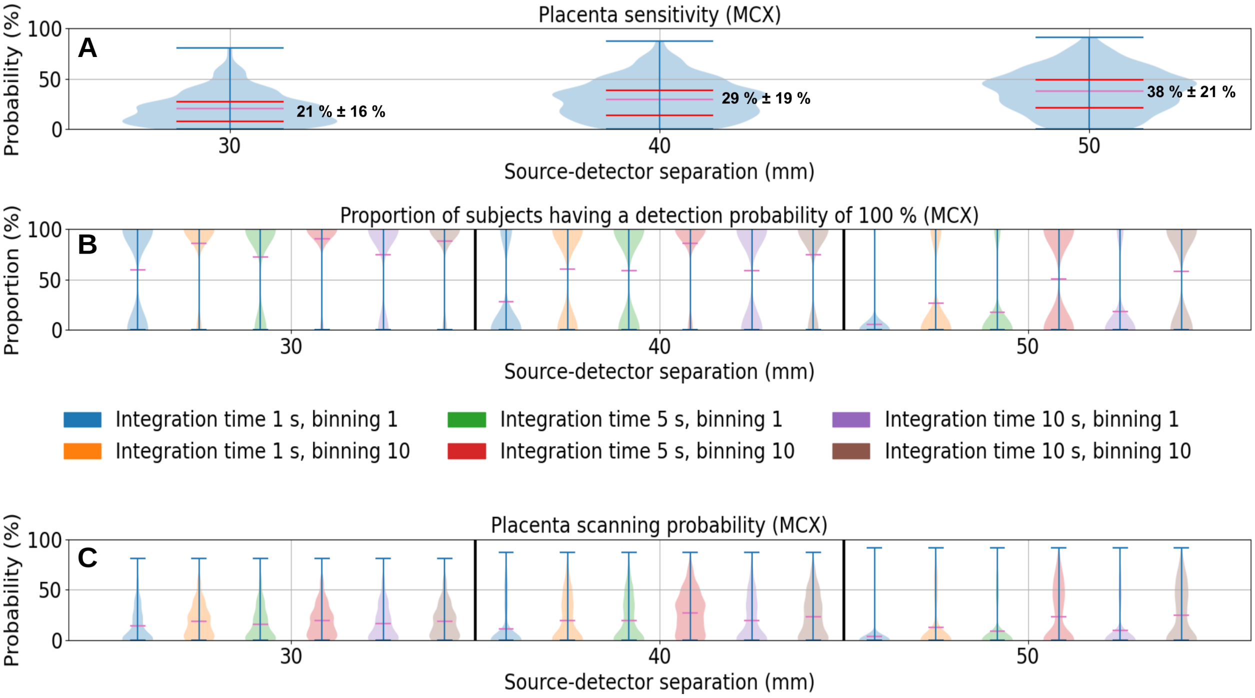

Fig S11 Placenta sensitivity (A), proportion of subjects having a detection probability of 100% (B) and placenta scanning probability (C). The distributions have been simulated for a Mini CYRIL device at 780 nm with 142 healthy subjects. Data have been calculated with Monte Carlo simulations (MCX). Horizontal magenta lines indicate the mean values of the distribution; red lines indicate the 25th and 75th percentiles. NIRS signals have been simulated based on clinical measurements of tissue thickness and skin tones. Subject tissue thickness have been measured with ultrasound imaging and the skin tones of the participants were estimated with the Fitzpatrick scale. For the calculation of the probabilities, total haemoglobin values for muscle and placenta layers were fixed to 35 μM. Oxygen saturation for the muscle and placenta layers was fixed to 60% and 80%, respectively. Absorption and scattering coefficients of the simulated maternal abdomen are listed below: $\mu_a^{\text{skin}} = 0.040$, 0.088 and 0.144 mm$^-1$ for a melanosome volume fraction of 2.55, 15.5 and 30.5%, respectively. $\mu_s^{\text{skin}} = 14.38$ mm^{-1}, $\mu_a^{\text{Adipose tissue}} = 0.002$ mm^{-1}, $\mu_s^{\text{Adipose tissue}} = 13.64$ mm^{-1}, $\mu_a^{\text{Muscle}} = 0.0089$ mm^{-1}, $\mu_s^{\text{Muscle}} = 8.61$ mm^{-1}, $\mu_a^{\text{Placenta}} = 0.0085$ mm^{-1}, $\mu_s^{\text{Placenta}} = 8.81$ mm^{-1}.

		Placental sensitivity	Detection probability	Scanning probability
Mean (%)	MCX	29.49	56.12	16.86
	Redbird	26.10	54.16	13.20
Std (%)	MCX	20.44	49.61	21.38
	Redbird	16.63	49.82	16.88

Table S1 Mean and standard deviation of the distribution of placental sensitivity, detection probability and scanning probability obtained with Redbird and Monte Carlo simulations (MCX). Data have been estimated with a Mini CYRIL device at 780 nm on 142 healthy subjects, see Fig. 11 in the manuscript and Fig. SS11. Metrics are indicated for all acquisition configuration.

	Placental sensitivity	Detection probability	Scanning probability
Mean (Redbird - MCX) (%)	4.18	6.69	4.39
Std (Redbird - MCX) (%)	4.08	24.97	10.46

Table S2 Mean and standard deviation of the absolute differences between distributions obtained with Redbird and Monte Carlo simulations (MCX). Data have been estimated with a Mini CYRIL device at 780 nm on 142 healthy subjects, see Fig. 11 in the manuscript and Fig. SS11. Metrics are indicated for all acquisition configuration.

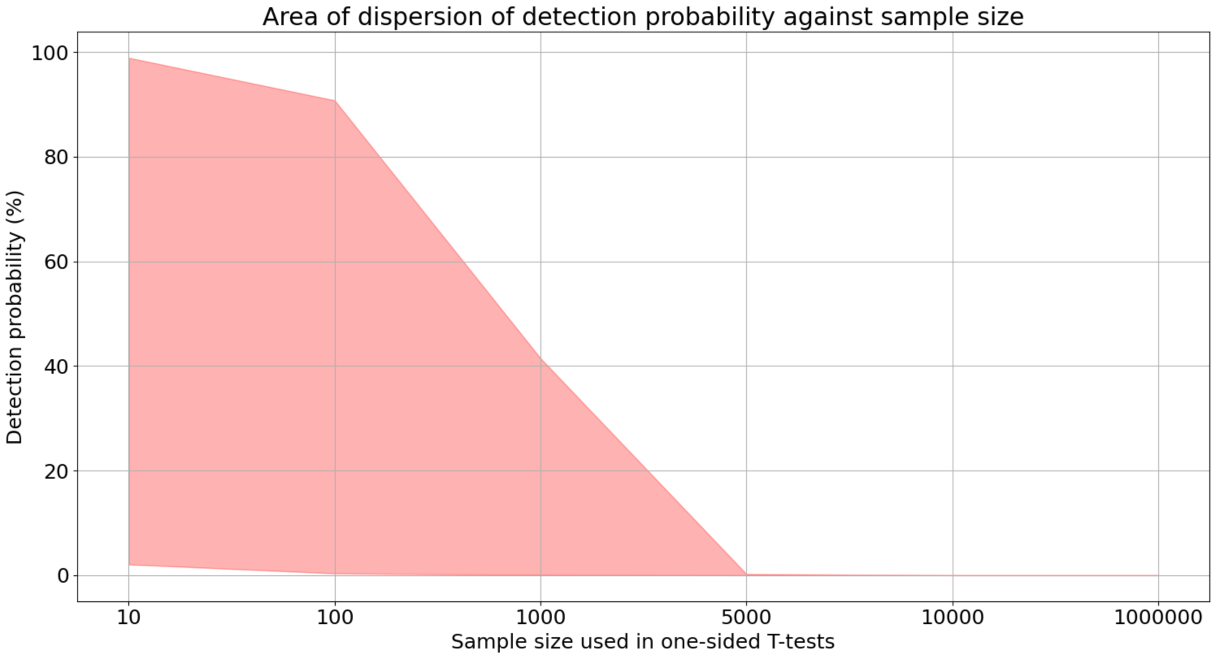

Fig S12 Area of dispersion of the detection probability as a function of the sample size (the calculation has been repeated 20 times). The diffuse reflectance value has been simulated for a placenta depth of 16 mm, a melanosome volume fraction of 30.5%, muscle and placenta blood volume of 25 μM and 35 μM, respectively, muscle and placenta $SatO_2$ of 60% and 80%, respectively.

			780 nm	840 nm	890 nm
Skin	$f_{mel} = 2.55$ %	μ_a (mm^{-1})	0.0406	0.0321	0.0270
	$f_{mel} = 15.5$ %	μ_a (mm^{-1})	0.0887	0.0706	0.0593
	$f_{mel} = 30.5$ %	μ_a (mm^{-1})	0.1443	0.1152	0.0968
		μ_s (mm^{-1})	14.3840	12.9463	11.9251
Adipose tissue		μ_a (mm^{-1})	0.0022	0.0035	0.0054
		μ_s (mm^{-1})	13.6470	12.9840	12.4892
Muscle	$C_{HbT} = 25\mu M$, $SatO_2 = 60$ %	μ_a (mm^{-1})	0.0069	0.0083	0.0103
	$C_{HbT} = 35\mu M$, $SatO_2 = 60$ %	μ_a (mm^{-1})	0.0089	0.0103	0.0126
		μ_s (mm^{-1})	8.6121	8.0409	7.6217
Placenta	$C_{HbT} = 15\mu M$, $SatO_2 = 80$ %	μ_a (mm^{-1})	0.0049	0.0068	0.0088
	$C_{HbT} = 25\mu M$, $SatO_2 = 80$ %	μ_a (mm^{-1})	0.0067	0.0090	0.0114
	$C_{HbT} = 35\mu M$, $SatO_2 = 80$ %	μ_a (mm^{-1})	0.0085	0.0112	0.0139
	$C_{HbT} = 50\mu M$, $SatO_2 = 80$ %	μ_a (mm^{-1})	0.0113	0.0145	0.0176
		μ_s (mm^{-1})	8.8147	7.9307	7.3031

Table S3 Absorption and scattering coefficients of skin, adipose tissue, muscle and placenta layers used in the study.
